# Supplementary material for: Lmo2 expression defines tumor cell identity during T‐cell leukemogenesis
Source: EMBO J. 2018 Jun 7;37(14):e98783. doi: 10.15252/embj.201798783 (PMC6043907; doi:10.15252/embj.201798783)

APPENDIX

Table of Contents:

Appendix Supplementary Methods Page 1

Appendix Supplementary Figure Legends Page 13

References Page 21

Appendix Supplementary Figures S1-S9 Page 25

Appendix Supplementary Methods:

Generation of mouse strains

All animal work has been conducted in accordance with national and international guidelines on animal care and was approved by the Bioethics Committee of University of Salamanca and by the Bioethics Subcommittee of Consejo Superior de Investigaciones Cientificas (CSIC). The Rosa26-Lmo2 vector was generated as follow: the mouse *Lmo2* cDNA clone was subcloned into the Rosa26UA plasmid using AscI site. This plasmid contained the Loxp-pgk-Neo-tPA-Loxp- AscI-FheI-IRES-EGFP within the Rosa26 homology arms, and the final targeting construct contained *Loxp-pgk-Neo-tPA-Loxp-Lmo2-IRES-EGFP*.

The G4 mouse ES cell line (George et al, 2007) a generous gift of Dr Andras Nagy and Dr. Marina Gertsenstein, was used to modify the Rosa26 locus by homologous recombination at the Servicio de Transgénesis CNB-CBMSO UAM/CSIC (Madrid), where chimeric mice were also generated. Heterozygous Rosa26-Lmo2 animals were obtained after mating chimera with C57BL/6J females. The Rosa26-Lmo2 mice were bred to *Sca1-Cre* (Mainardi et al, 2014), *Mb1-Cre* (Hobeika et al, 2006) or *Aid-Cre* mice (Crouch et al, 2007) to generate

Rosa26-Lmo2+Sca1-Cre, *Rosa26-Lmo2+Mb1-Cre*, *Rosa26-Lmo2+Aid-Cre* mice, respectively.

The *Sca1-Lmo2* vector was generated by inserting the *TdTomato–IRES*-mouse *Lmo2* cassette into the ClaI site of the pLy6 vector (Miles et al, 1997). The transgene fragment was excised from its vector by restriction digestion with NotI, purified and injected (2 ng/uL) into CBAxC57BL/6J fertilized eggs. Transgenic mice with high copy-number (needed to generate a mosaic of *Lmo2* expression in thymus T-cells) were identified by Southern blot analysis of tail snip DNA after EcoRI digestion, using *Lmo2* cDNA to detect the transgene. Two independent transgenic lines were generated and analyzed and they demonstrated similar phenotypic features. Upon clinical manifestations of disease, mice were sacrificed and subjected to standard necropsy procedures. All major organs were examined under the dissecting microscope. Tissue samples were taken from homogenous portions of the resected organ and fixed immediately after excision. Differences in survival of transgenic and control WT mice were analyzed using the log-rank (Mantel-Cox) test.

Flow cytometry

Nucleated cells were obtained from whole mouse bone marrow (flushing from the long bones), peripheral blood, thymus, lymph nodes and spleen. In order to prepare cells for flow cytometry, contaminating red blood cells were lysed with RCLB lysis buffer and the remaining cells were washed in PBS with 1% FCS. After staining, all cells were washed once in PBS with 1% FCS. The samples and the data were acquired in an AccuriC6 Flow Cytometer (Becton Dickinson) and analyzed using the FlowJo software (TreeStar). Specific fluorescence of

FITC and APC excited at 488 nm (0.4 W) and 633 nm (30 mW), respectively, as well as known forward and orthogonal light scattering properties of mouse cells were used to establish gates. Nonspecific antibody binding was prevented by preincubation of cells with CD16/CD32 (2.4G2) Fc-block solution (BD Biosciences). For each analysis, a total of at least 100,000 viable cells were assessed.

The following antibodies were used for flow cytometry: anti-B220 (RA3-6B2), CD3Ɛ (145-2C11), CD4 (RM4-5, 1:250), CD8a (53-6.7, 1:250), CD11b/Mac1 (M1/70), CD19 (1D3), Ly-6G and Ly-6c/Gr1 (RB6-8C5), CD44 (IM7), IgD (11-26c.2a), CD23 (B3B4), CD25 (PC61), CD117 (2B8), IgM (II/41), CD21 (7G6), CD45 (30F-11) and Sca1 (E13-161.7) antibodies. FACS definition of different developmental stages was performed as follows thymus double negative DN1 T cells ($CD44^{+}$ $CD25^{-}$ Lin^{-}(Lin: CD4, CD8a)), thymus double negative DN2 T cells ($CD44^{+}$ $CD25^{+}$ Lin^{-}), thymus double negative DN3 T cells ($CD44^{-}$ $CD25^{+}$ Lin^{-}), thymus double negative DN4 T cells ($CD44^{-}$ $CD25^{-}$ Lin^{-}), thymus double positive T cells ($CD4^{+}$ $CD8^{+}$), peripheral CD4 T cells ($CD4^{+}$ $CD8^{-}$), peripheral CD8 T cells ($CD4^{-}$ $CD8^{+}$), BM HSC (Lin^{-} $Sca1^{hi}$ c-Kit^{hi}), BM pro-B cells ($CD19^{+}$c-Kit^{+}), BM pre-B cells ($B220^{+}CD25^{+}IgM^{-}$), BM and spleen immature B-cells ($B220^{+}IgM^{hi}IgD^{-}$), BM recirculating B-cells ($B220^{hi}IgD^{hi}IgM^{lo}$), peripheral transitional B-cells ($B220^{+}IgM^{hi}IgD^{hi}$), peripheral mature-B cells ($B220^{+}IgM^{lo}IgD^{hi}$), marginal zone (MZ) B-cells ($B220^{+}CD21^{hi}CD23^{lo}$), peripheral follicular (FO) B-cells ($B220^{+}CD21^{int}CD23^{hi}$), and peripheral myeloid cells ($Gr1^{hi}$ $Mac1^{hi}$ (Cobaleda et al, 2007; Delogu et al, 2006; Schebesta et al, 2007). All antibodies were purchased from BD Biosciences. All antibodies were used at a 1:100 dilution unless otherwise indicated.

Histology and Immunohistochemistry (IHC)

Animals were sacrificed by cervical dislocation; tissue samples were formalin-fixed and included in paraffin. Tissue samples were taken from homogenous portions of resected organs by the pathologist and fixed immediately after excision. Samples of each organ were processed into paraffin, sectioned and examined histologically including hematoxylin and eosin and immunohistochemical stainings. Transgenic mice samples were sectioned, dewaxed, and heated in 10 mmol/L sodium citrate buffer for 30 min. Slides were incubated with primary antibodies. The antibodies used included: PAX5 *(clone* 24/PAX5, dilution 1:100, BD Biosciences, San Jose, CA), *B220 (clone RA3-6B2, dilution 1:50, Santa Cruz Biotechnology, Inc., Santa Cruz, CA), TdT (dilution 1.50, Supertechs INC), LMO2 (clone 1A9-1, dilution 1:150), and CD3 (clone SP7, dilution 1:50, Abcam, Cambridge, MA)*. Samples were centrally reviewed by a panel of pathologists and diagnosed using uniform criteria based on clinical, histological, immunophenotypical, and molecular characteristics. For comparative studies, age-matched mice were used.

Real- time PCR quantification (Q-PCR) of *Cdkn2a* and *Lmo2*

We analyzed expression of *Lmo2* and *Cdkn2a* in sorted subpopulation from wild type and transgenic hematopoietic cells by Q-PCR as follows: cDNA was synthesized using reverse transcriptase (Access RT-PCR System; Promega, Madison, WI) and genomic DNA was removed by DNAase treatment (Roche, 04 716 728 001). Real-time PCR reactions were performed in an Eppendorf MasterCycler Realplex machine. Commercially available assays for quantitative

PCR from IDT (Integrated DNA Technologies) were used: *Lmo2* (Assay ID: Mm.PT.56a.21550807), *Cdkn2a* (Assay ID: Mm.PT.49a.5632963) and *Gadph* (Assay ID: Mm.PT.39a.1). Probes were specifically designed to prevent detection of genomic DNA by PCR. Measurement of GAPDH gene product expression was used as an endogenous control. All samples were run in triplicate. The comparative CT Method ($\Delta\Delta$Ct) was used to calculate relative expression of the transcript of interest and a positive control.

Immunoglobulin rearrangements.

Immunoglobulin rearrangements were amplified by PCR using the primers below. Cycling conditions consisted of an initial heat-activation at 95ºC followed by 31-37 cycles of denaturation for 1 min at 95ºC, annealing for 1 min at 65ºC, and elongation for 1 min 45 s at 72ºC. This was followed by a final elongation for 10 min at 72ºC. To determine the DNA sequences of individual V(D)J rearrangements, the PCR fragments were isolated from the agarose gel and cloned into the pGEM-Teasy vector (Promega); the DNA inserts of at least ten clones corresponding to the same PCR fragment were then sequenced. The following primer pairs were used:

V_HJ558	forward	CGAGCTCTCCARCACAGCCTWCATGCARCTCARC
	reverse	GTCTAGATTCTCACAAGAGTCCGATAGACCCTGG
V_H7183	forward	CGGTACCAAGAASAMCCTGTWCCTGCAAATGASC
	reverse	GTCTAGATTCTCACAAGAGTCCGATAGACCCTGG
V_HQ52	forward	CGGTACCAGACTGARCATCASCAAGGACAAYTCC
	reverse	GTCTAGATTCTCACAAGAGTCCGATAGACCCTGG
DH	forward	TTCAAAGCACAATGCCTGGCT
	reverse	GTCTAGATTCTCACAAGAGTCCGATAGACCCTGG
Cμ	forward	TGGCCATGGGCTGCCTAGCCCGGGACTT
	reverse	GCCTGACTGAGCTCACACAAGGAGGA

TCR rearrangements.

Immunoglobulin rearrangements were amplified by PCR using the primers below. Cycling conditions consisted of an initial heat-activation at 95ºC followed by 35 cycles of denaturation for 1 min at 95ºC, annealing for 1 min at 57ºC, and elongation for 1 min 45 s at 72ºC. This was followed by a final elongation for 10 min at 72ºC. To determine the DNA sequences of individual V(D)J rearrangements, the PCR fragments were isolated from the agarose gel and cloned into the pGEM-Teasy vector (Promega); the DNA inserts of at least ten clones corresponding to the same PCR fragment were then sequenced. The following primer pairs were used:

$D_{\beta}2$	forward	GTAGGCACCTGTGGGGAAGAAACT
$V_{\beta}2$	forward	GGGTCACTGATACGGAGCTG
$V_{\beta}4$	forward	GGACAATCAGACTGCCTCAAGT
$V_{\beta}5.1$	forward	GTCCAACAGTTTGATGACTATCAC
$V_{\beta}8$	forward	TGGCCATGGGCTGCCTAGCCCGGGACTT
$V_{\beta}14$	forward	CTTCTACCTCTGTGCCTGGAGT
$J_{\beta}2$-Rev	Reverse (common to all)	TGAGAGCTGTCTCCTACTATCGATT

Array comparative genomic hybridization (aCGH)

Whole genome analysis was conducted using an *M.musculus* whole genome 4x180k oligonucleotide array-CGH (AMADID 27411, Agilent Technologies), following standard protocol. Microarray data were extracted and visualized using Feature Extraction software v10.7 and Agilent Genomic Workbench v5.0 (Agilent Technologies). Copy number altered regions were detected using ADM-2 (set as 6) statistic with a minimum number of 5 consecutive probes. Genomic build mm9 was used for the experiment.

Gene expression microarray analysis of murine tumors

Tumoral and normal thymuses were harvested from 11 *Sca1-Lmo2* mice and 4 control littermate wild-type mice, respectively. Cells were not sorted prior to RNA extraction for this analysis. Total RNA was isolated using TRIzol (Life Technologies) followed by Rneasy Mini-Kit (Qiagen) purification following the manufacturer's RNA Clean-up protocol with the optional On-column DNase treatment. The integrity and the quality of the RNA were verified by electrophoresis and its concentration measured by the NanoDrop 1000 (Thermo Scientific) and Agilent 2100 Bioanalyzer (Agilent Technologies). Samples were analyzed using Affymetrix Mouse Gene 1.0 ST arrays. Briefly, the robust microarray analysis (RMA) algorithm was used for background correction, intra- and inter-microarray normalization, and expression signal calculation (Bolstad et al, 2003; Irizarry et al, 2003a; Irizarry et al, 2003b). Once the absolute expression signal for each gene (i.e., the signal value for each probe set) was calculated in each microarray, significance analysis of microarray (SAM) (Tusher et al, 2001) method was used to calculate significant differential expression and to find gene probe sets that characterized the tumor T –cells from *Sca1-Lmo2* mice compared with normal T-cells from WT mice. A cutoff of FDR < 0.05 (Benjamini et al, 2001) was used for the differential expression calculations. All the analyses were performed using R and Bioconductor (Gentleman et al, 2004).

Enrichment analysis

Differentially expressed genes were tested for enrichment of genes associated with curated gene sets C2 collection from MSigDB (Subramanian et al, 2005)

looking for molecular signatures within *Sca1-Lmo2* mice. Gene Set Enrichment Analysis (GSEA) was performed using a weighted enrichment statistic, signal-to-noise ratio ranking metric, and corrected for multiple hypothesis testing (MHT) using 1000 permutations to provide an MHT-corrected false discovery rate (FDR) q-value.

The data discussed in this publication and compared with, are deposited in the Molecular Signatures Database (MSigDB) of the MIT Broad Institute and are accessible through the following names: WONG_EMBRYONIC_STEM_CELL_CORE, MUELLER_PLURINET and DANG_MYC_TARGETS_UP and HALLMARK_NOTCH_SIGNALING.

FISH analysis

Fluorescence in situ hybridization (FISH) was performed with two BAC clones that flank the 5' end (CY3-labelled RP11-98C11 in red) and the 3' end (FITC-labeled RP11-60G13 in green) of the *LMO2* gene as described previously (Konig et al, 2002).

Mouse exome library preparation and next generation sequencing

Sample acquisition: The AllPrep DNA/RNA Mini Kit (Qiagen, Hilden, Germany) was used to purify DNA according to the manufacturer’s instructions.

Exome library preparation and next generation sequencing: Exome library preparation was performed using the Agilent SureSelectXT Mouse All Exon kit with modifications adapted from Fisher et al., 2011 (Fisher et al, 2011). Briefly, we added SPRI beads to the original protocol and reduced the size of the reaction to 0.5 µl in order to be able to use PCR tubes for subsequent steps.

Furthermore, we reduced the volume for washing. We minimized sample loss and optimized sample processing by reducing sample handling. We therefore just added freshly prepared 20% PEG/2.5 M NaCl (Sigma) instead of elution of samples from the SPRI beads for library preparation. Targeted capture by hybridization to an RNA library was performed according to the manufacturer's protocol. Purification and enrichment of the captured library were achieved by binding to MyOne Streptavidin T1 Dynabeads (LifeTechnolgies) and off-bead PCR amplification in the linear range. 2x100 bp sequencing with a 6 bp index read was performed using the TruSeq SBS Kit v3 on the HiSeq 2500 (Illumina).

Data analysis: Fastq files were generated by using BcltoFastq 1.8.4 (Illumina). BWA version 0.7.4 (Li & Durbin, 2010)was used to align sequence data to the mouse reference genome (GRCm38.71). Conversion steps were carried out using Samtools (Li & Durbin, 2009; Li et al, 2009) followed by removal of duplicate reads (http://broadinstittute.github.io/picard). Local realignment around indels, SNP-calling, annotation and recalibration were facilitated by GATK 2.4.9 (DePristo et al, 2011) Mouse dbSNP138 and dbSNP for the used mouse strains were used as training datasets for recalibration. Resulting variation calls were annotated by Variant Effect Predictor (McLaren et al, 2010) using the Ensembl database (v70) and imported into an in-house MySQL database to facilitate automatic and manual annotation, reconciliation and data analysis by complex database queries. Loss of function prediction scores for PolyPhen2 (Adzhubei et al, 2010) and SIFT (Kumar et al, 2009) were extracted from this Ensemble release.

Somatic calls were produced using MuTect (Cibulskis et al, 2013). Only entries with at least 9% difference in allele frequency between tumor and normal were

kept for further analysis. Cancer-related genes were determined by translating the cancer gene consensus from COSMIC (Forbes et al, 2015) using ENSEMBL's biomart (Smedley et al, 2015).

Sequencing.

Mutations were validated using Sanger sequencing on a 3130 Genetic Analyzer (Applied Biosystems). List of primers used for Sanger sequencing:

Mouse: Notch1	For: TTGGATGCCCTGGAATTAGA<br>Rev: TAAGGAAAAGGGGTGCTGTG<br>For: AAACAGCCCACAACAGCAG<br>Rev: AGGTATGGGTTTGAGGTCCA<br>For: ATGGAGGATGGCAGTGATGT<br>Rev: GGGGGCTATGAATTTCACC<br>For: GTTGTCCACAGGGGAGGAG<br>Rev: GCCAGTACAACCCACTACGG
Mouse: Kras	For: TGGTTCCCTAACACCCAGTT<br>Rev: GAAGATGAAAGTACTGGTTTCCA
Mouse: Nras	For: CACAGATATAAATTCACCTGCCC<br>Rev: ATACTTTAGTATAGTTTCAGAG
Mouse: Cdh11	For: AAATGAGTCCATGTGTCAAAAC<br>Rev: TGGTCAGGGCAGGAGAAGCTAG
Mouse: Cd1d1	For: CAAATAGGGGGCAGGTGTCATT<br>Rev: ACCCTTATGGTTTTACTTCTGTC
Mouse:Sept6	For: GTAGTTCTGTCTGTCAAGGCTG<br>Rev: TCCTGCCCTGCCCCACCCCACC
Mouse: Hspa1l	For: AGAGGAGATCTCATCCATGG<br>Rev: GCTTCCTCTTGAACTCCTCC
Mouse: Stat5a	For:CTCCAATACTGGGAAATGTGG<br>Rev:CAAAATGCTCTGAGCGACTG
Mouse: IKZF1	For:AATTGACCCAGCCAGTGAAG<br>Rev:ACCACATAGGAGGCCAACAC
Mouse: Rin3	For:AGCAAAGCCAGGACACAGAG<br>Rev:GAGAGGAAGGCATGGAAGAC
Mouse: TBX3	For:AAACTATGCGTCGCTGTTGA

	Rev:ATCTCTGTACCCCGCTTGTG
Mouse: Abca13	For:TATGGGTCCTGACACCAAC<br>Rev:CTGCAGCCCTGGTAGGAGTA
Mouse: Cdkn2a	For:GATGTCTTGATGTCCCCGCT<br>Rev:CCGGCGATGTTCTACAGGAG
Mouse: Abcb1b	For:TCTGCTGGAAACAATCTGTCA<br>Rev:TGGGGCAGAGTCATCCTATC
Mouse: Itga2b	For:TTGTCTCCGCCTACAAGAGG<br>Rev:CAGGGGATGAGACACGAAAC
Mouse: Setbp1	For:CATTGGCTTTGTGGAACAGA<br>Rev:CAGCACTGGACACAGGCTAA
Mouse: Abca8b	For:AATGTCCACTTTCTCGGGGC<br>Rev:AGGATCCGAGGGGATCAGTG
Mouse: Pcdh15	For:TTGCGTGAGGCTTTTACCCA<br>Rev:GTTGTCTCGGACTCGCTGAA
Mouse: Flt4	For:GGAGAGCATGGAGGTAGCAG<br>Rev:GTGCAGCGAGTTAGGGACTC

Human: NOTCH1	For: AAAAAGGCTCCTCTGGTCGG<br>Rev: GAATGGTCAATGCGAGTGGC<br>For: GGATGGGGCCACACTTACTC<br>Rev: CCTCACCATGTCCTGACTGT<br>For: GAGAGTTGCGGGGATTGACC<br>Rev: ATGCAGTTCTAAGGCTCTGC<br>For: CAGAGGACCTTGATGGGCTG<br>Rev: CCCCTAGGGTTGAGCAGAAG
Human: KRAS	For: CCCTGACATACTCCCAAGGA<br>Rev: CGATACACGTCTGCAGTCAA
Human: NRAS	For: GCAGCTAATAAAAATGAACTGTTCTC<br>Rev: TAAGGATGGGGGTTGCTAGA
Human: FBXW7	For: TCAAACAGGAAGCTGACAACA<br>Rev: AACTTTACTCCAAATTTTAATGAGCTA<br>For: CATGTGGCCTTTTGTGTCTG<br>Rev: TGTGATCTCTGGGAAGAAAGG
Human: MTOR	For: AAAGTGTGCTCAGATTTTATG<br>Rev: CGGACATAGAGGAAGGATTGC

RNA Sequencing

RNA Sequencing libraries were generated from 500 ng of total RNA using the Truseq RNA sample prep kit (Illumina) from the blast cells obtained different mouse models employed in the study, including cells from healthy thymus as a control (wt). Later the libraries were subjected to 2×100 bp paired-end sequencing using HiSeq2000 instrument (Illumina). The RNA-seq data was aligned against the mouse reference genome (mm10/GRCm38.83) with TopHat 2.1.0 (Kim et al, 2013), and FPKM values per gene were calculated with the R/Bioconductor package bamsignals (Mammana & and Helmuth, 2016). Genes with FPKM values > 1 in less than three samples were excluded from the analysis. We selected the 500 genes with the highest variance over all samples, transformed the corresponding FPKM values into standard scores (**Table EV5**) and visualized the results with the R/Bioconductor package gplots' heatmap.2 function (row dendrogram, clustering method 'ward.D') (Warnes et al, 2016).

Differential analysis between the mouse groups *Rosa26-Lmo2+Sca1-Cre* and *Sca1-Lmo2* against the wild type group was conducted with DESeq2 (Love et al, 2014), with a minimum adjusted p-value of 0.05. Signatures of the 100 most significantly up- and downregulated human homologue genes per differential analysis were then tested for enrichment with the Broad Institute's GSEA tool (**Table EV6**) (Mootha et al, 2003; Subramanian et al, 2005), against a human childhood T-ALL set with corresponding wild-type thymus control samples (Ng et al, 2014).

Appendix Supplementary Figure Legends

Appendix Figure S1: Conditional expression of the *Lmo2* oncogene in the mouse HSCs.

A) Schematic showing the *Lmo2* cDNA cloned into a vector designed for targeting into the mouse *Rosa26* locus. neo-R, neomycin resistance cassette; polyA, polyadenylation stop sequence; IRES, internal ribosome entry site; *eGFP*, enhanced green fluorescent protein. Cre-mediated recombination excises the stop sequence and initiates expression of the fusion oncogene and *eGFP*. To prevent transcription of the fusion gene and eGFP from the *Rosa26* promoter, a neomycin resistance cassette and poly-adenylation stop signal flanked by loxP sites were inserted between the promoter and the *Lmo2-IRES-eGFP* sequence. In the absence of Cre, neither Lmo2 nor eGFP are expressed, due to a STOP cassette in front of *Lmo2* that has to be removed by Cre recombinase action to allow for their expression.

B) Mouse embryonic stem cells confirmed to bear the targeted allele were injected into blastocysts to generate chimeras that were then bred to generate progeny with a germline-transmissible conditional allele of Lmo2 (designated *Rosa26-Lmo2*). Identification of *Rosa26-Lmo2* mice by Southern analysis of tail snip DNA after EcoRI digestion.

C) GFP expression in peripheral blood hematopoietic cells from preleukemic *Rosa26-Lmo2+Sca1-Cre* mice showing that GFP is expressed in all hematopoietic cells.

D) Representative hematopoietic composition in *Rosa26-Lmo2+Sca1-Cre*

preleukemic mice (4 months). No major abnormalities were detected in differentiated myeloid cells, B cells or T cell populations.

E) Percentage of T-cells analyzed by flow cytometry at different developmental stages (DN –double negative- T cells, DP –double positive- T cells, CD8 T cells and CD4 T cells) in the thymuses of preleukemic *Rosa26-Lmo2+Sca1-Cre* mice (n=4, 4 months old) compared to control littermate *wild-type* mice (n=4, 4 months old). Preleukemic *Rosa26-Lmo2+Sca1-Cre* mice only show significant increase in CD4+ T-cells (p value=0.0286; Mann-Whitney test).

Appendix Figure S2: **GFP expression in different hematopoietic tissues of *Rosa26-Lmo2+Sca1-Cre* preleukemic mice**.

A-D) Flow cytometric analysis of thymus **(A)**, spleen **(B)**, bone marrow **(C)** and peripheral blood **(D)** of 4 months old preleukemic *Rosa26-Lmo2+Sca1-Cre* mice. Representative plots of cell subsets close to the tracking of the GFP marker for *Lmo2* transgene expression are shown and compared to aged-matched control littermate *wild-type* mice.

Appendix Figure S3: T-ALL development in *Rosa26-Lmo2+Sca1-Cre* mice

A) Example of splenomegaly observed in *Rosa26-Lmo2+Sca1-Cre* mice studied. A spleen from a control *wild-type* littermate mouse is shown for reference.

B) Hematoxylin and eosin staining showing infiltration of spleen and liver from *Rosa26-Lmo2+Sca1-Cre* leukemic mice. Tissues from a control littermate *wild type* mouse are shown for reference. Scale bar represents 500μm (=10X) for large panels and 100μm (=40X) for inset.

C) Flow cytometric analysis of tumor T-cells in thymus, spleen, lymph nodes, bone marrow and peripheral blood of diseased *Rosa26-Lmo2+Sca1-Cre* mice. These tissues also exhibited an aberrant immunophenotype, mimicking that seen in the thymus. Same tissues from a control littermate *wild-type* mouse are shown for reference**.**

Appendix Figure S4: Expression and activity of *Lmo2* in Sca1-Lmo2 T cells.

A) Schematic representation of the genomic structure of the mouse *Sca1* locus and the *Sca1-Lmo2* transgenic vector used in this study. NotI sites used to excise the transgene fragments and EcoRI sites used to examine Southern blots are indicated. *Lmo2* expression within HSPCs was achieved by placing a *Lmo2* cDNA linked to the fluorescent *TdTomato* cDNA by an internal ribosome entry under the control of the promoter for the HSPC-specific gene, stem-cell-antigen 1 (Sca1).

B) Relative expression of *Lmo2* in sorted-purified T-cells at different developmental stages (DN –double negative- T cells, DP –double positive- T cells, CD8 T cells and CD4 T cells) in the thymuses of preleukemic Sca1-Lmo2 mice compared to control littermate *wild-type* mice. Total bone marrow of a wild-type control mouse was used as a positive control. The fold change in each group, calculated as 2–ΔΔCt sample, was compared. Bars represent the mean and the standard deviation.

C-G) Flow cytometric analysis of thymus **(C)**, spleen **(D)**, bone marrow **(E)**, peripheral blood **(F)** and lymph nodes **(G)** of 3 months old preleukemic *Sca1-Lmo2* mice. Representative plots of cell subsets close to the tracking of the

TdTomato marker for *Lmo2* transgene expression are shown and compared to aged-matched control littermate *wild-type* mice.

H) Percentage of T-cells at different developmental stages (DN –double negative- T cells, DP –double positive- T cells, CD8 T cells and CD4 T cells) in the thymuses of preleukemic *Sca1-Lmo2* mice (n=22, 3-4 months old) compared to control littermate *wild-type* mice (n=10, 3-4 months old) analyzed by flow cytometry. A significant increase in DN T cells and CD8 T cells, respectively, can be observed in preleukemic *Sca1-Lmo2* mice. Bars represent the mean and the standard error of the mean. The statistical significance is denoted with an asterisk (analyzed by the Mann Whitney Test).

I) ARF expression in thymuses control littermate *wild-type* mice and in thymuses of preleukemic *Sca-Lmo2* mice.

J) Tracking of the TdTomato marker for *Lmo2* transgene expression during T-cell development in the thymus shows a mosaic of *Lmo2* expression in thymus T-cells. For gating scheme, a representative plot of cell subsets from the thymus is shown. Percentage of TdTomato positive and TdTomato negative cells within the different developmental stages (DN –double negative- T cells, DP –double positive- T cells, CD8 T cells and CD4 T cells) in the thymuses of 20 preleukemic *Sca1-Lmo2* mice analyzed by flow cytometry is shown. Bars represent the mean and the standard deviation. Flow cytometric images are representative of the analysis of 20 preleukemic *Sca1-Lmo2* mice.

Appendix Figure S5: Expression of the *Lmo2* oncogene in hematopoietic progenitor cells (HSPCs) causes aggressive malignancy of mature T-cells.

A) Thymomas in *Sca1-Lmo2* mice bearing T-ALL (n=17) were formed by a

significant increase in the number of thymocytes compared to preleukemic *Sca1-Lmo2* mice (n=16) and to control littermate *wild-type* mice (n=10), respectively.

B) Flow cytometric analysis of tumor T-cells in thymus, spleen, lymph nodes, bone marrow and peripheral blood of diseased *Sca1-Lmo2* mice. These tissues also exhibited an aberrant immunophenotype, mimicking that seen in the thymus. Same tissues from a control littermate *wild-type* mouse are shown for reference.

C) Examples of splenomegaly observed in leukemic *Sca1-Lmo2* mice. A spleen from a control littermate *wild-type* mouse is shown for reference.

D) Hematoxylin and eosin staining of control littermate wild-type mice and T-ALL-bearing *Sca1-Lmo2* mice showing infiltrating blast cells in thymus, small intestine, kidney, liver, lung and spleen. Loss of normal architecture resulting from effacement with cells morphologically resembling lymphoblasts can be shown. Tissues from a control littermate *wild type* mouse are shown for reference. Images are representative of 3 replicates. Scale bar represents 500μm (=10X) for large panels and 100μm (=40X) for inset.

E) T cell receptor (TCR) clonality in Sca1-Lmo2 mice. PCR analysis of TCR gene rearrangements in infiltrated thymus of diseased *Sca1-Lmo2* leukemic mice. Sorted double positive (DP) T cells from the thymus of healthy mice serve as a control for polyclonal TCR rearrangements. Leukemic T cells show an increased clonality within their TCR repertoire (indicated by code number of each *Sca1-Lmo2* mouse analyzed).

Appendix Figure S6: Characterization of preleukemic T cells lacking Lmo2 expression.

A) Genes significantly induced and repressed within Tomato-positive T cells of preleukemic *Sca1-Lmo2* mice versus *wild-type* littermates.

B) Genes significantly induced and repressed within Tomato-negative T cells of preleukemic *Sca1-Lmo2* mice versus *wild-type* littermates.

C) Genes significantly induced and repressed within Tomato-positive T cells of preleukemic *Sca1-Lmo2* mice versus Tomato-negative T cells of preleukemic *Sca1-Lmo2* littermates.

D) Genes significantly induced and repressed within Tomato-positive T cells of preleukemic *Sca1-Lmo2* mice versus tumoral T cells of leukemic *Sca1-Lmo2*.

E) Genes significantly induced and repressed within Tomato-negative T cells of preleukemic *Sca1-Lmo2* mice versus tumoral T cells of leukemic *Sca1-Lmo2.* The significance analysis of microarrays was defined by a FDR =1%. Each row represents a separate gene and each column a separate mRNA simple. The level of expression of each gene in each sample is represented using a red-blue color scale (genes upregulated are displayed in red color and those that are downregulated, are blue**.**

Appendix Figure S7: FISH analysis of patients with translocation t(11;14) positive T-ALL. A co-localized red/green signal defines the wild type *LMO2* allele, whereas separated red and green signals provide an indirect evidence for the presence of a gene region-disrupting translocation with a *TRD/LMO2* gene fusión (Konig et al, 2002). Shown are 3 representatives out of 8.

Appendix Figure S8: Leukemia development in Sca1-Lmo2+nu/nu mice.

A) Hematoxylin and eosin staining showing infiltration of lymph nodes, kidneys, pancreas, lung and liver from *Sca1-Lmo2+nu/nu* leukemic mice. Tissues from a control littermate *nu/nu* mouse are shown for reference.

B) Genes significantly induced or repressed in tumor cells of *Sca1-Lmo2+nu/nu* mice in comparison with *nu/nu* littermates, as determined by significance analysis of microarrays using an FDR 10%. Each row represents a separate gene and each column a separate mRNA simple. The level of expression of each gene in each sample is represented using a red-blue color scale (genes upregulated are displayed in red color and those that are downregulated, are blue).

C) Genes significantly induced or repressed in tumor cells of *Sca1-Lmo2+nu/nu* mice in comparison with *Sca1-Lmo2* tumor T cells, as determined by significance analysis of microarrays using an FDR 1%. Each row represents a separate gene and each column a separate mRNA simple. The level of expression of each gene in each sample is represented using a red-blue color scale (genes upregulated are displayed in red color and those that are downregulated, are blue).

Appendix Figure S9. T-ALL development through *Lmo2* expression in B cells.

A) GFP expression analyzed by FACS of bone marrow B cells, bone marrow myeloid cells and thymus T-cells from preleukemic *Rosa26-Lmo2+Mb1-Cre* mice showing that GFP is only detected in all B cells compartments.

B) Percentage of B-cells analyzed by flow cytometry at different developmental stages in the bone marrow of representative preleukemic *Rosa26-Lmo2+Mb1-Cre* mouse (n=4, 4 months old) compared to control *wild-type* littermate.

C) Hematoxylin and eosin staining showing blast infiltration of spleen, liver and thymus from *Rosa26-Lmo2+Mb1-Cre* leukemic mice. Tissues from a control littermate *wild type* mouse are shown for reference. Scale bar represents 500μm (=10X) for large panels and 100μm (=40X) for inset.

D) GFP expression of germinal center B cells in spleen from preleukemic immunized *Rosa26-Lmo2+Aid-Cre* mice showing that GFP is expressed in the germinal center B cell compartment.

E) GFP expression was not detected in bone marrow progenitor B cells, bone marrow myeloid cells and thymus T-cells from preleukemic *Rosa26-Lmo2+Aid-Cre* mice.

F) Hematoxylin and eosin staining showing blast infiltration of spleen, liver, kidney and lung from *Rosa26-Lmo2+Aid-Cre* leukemic mice. Scale bar represents 500μm (=10X) for large panels and 100μm (=40X) for inset.

References

Adzhubei IA, Schmidt S, Peshkin L, Ramensky VE, Gerasimova A, Bork P, Kondrashov AS, Sunyaev SR (2010) A method and server for predicting damaging missense mutations. *Nat Methods* **7:** 248-249

Benjamini Y, Drai D, Elmer G, Kafkafi N, Golani I (2001) Controlling the false discovery rate in behavior genetics research. *Behav Brain Res* **125:** 279-284

Bolstad BM, Irizarry RA, Astrand M, Speed TP (2003) A comparison of normalization methods for high density oligonucleotide array data based on variance and bias. *Bioinformatics* **19:** 185-193

Cibulskis K, Lawrence MS, Carter SL, Sivachenko A, Jaffe D, Sougnez C, Gabriel S, Meyerson M, Lander ES, Getz G (2013) Sensitive detection of somatic point mutations in impure and heterogeneous cancer samples. *Nat Biotechnol* **31:** 213-219

Cobaleda C, Jochum W, Busslinger M (2007) Conversion of mature B cells into T cells by dedifferentiation to uncommitted progenitors. *Nature* **449:** 473-477

Crouch EE, Li Z, Takizawa M, Fichtner-Feigl S, Gourzi P, Montano C, Feigenbaum L, Wilson P, Janz S, Papavasiliou FN, Casellas R (2007) Regulation of AID expression in the immune response. *J Exp Med* **204:** 1145-1156

Delogu A, Schebesta A, Sun Q, Aschenbrenner K, Perlot T, Busslinger M (2006) Gene repression by Pax5 in B cells is essential for blood cell homeostasis and is reversed in plasma cells. *Immunity* **24:** 269-281

DePristo MA, Banks E, Poplin R, Garimella KV, Maguire JR, Hartl C, Philippakis AA, del Angel G, Rivas MA, Hanna M, McKenna A, Fennell TJ, Kernytsky AM, Sivachenko AY, Cibulskis K, Gabriel SB, Altshuler D, Daly MJ (2011) A framework for variation discovery and genotyping using next-generation DNA sequencing data. *Nat Genet* **43:** 491-498

Edgar R, Domrachev M, Lash AE (2002) Gene Expression Omnibus: NCBI gene expression and hybridization array data repository. *Nucleic Acids Res* **30:** 207-210

Fisher S, Barry A, Abreu J, Minie B, Nolan J, Delorey TM, Young G, Fennell TJ, Allen A, Ambrogio L, Berlin AM, Blumenstiel B, Cibulskis K, Friedrich D, Johnson R, Juhn F, Reilly B, Shammas R, Stalker J, Sykes SM, Thompson J, Walsh J, Zimmer A, Zwirko Z, Gabriel S, Nicol R, Nusbaum C (2011) A scalable, fully automated process for construction of sequence-ready human exome targeted capture libraries. *Genome Biol* **12:** R1

Forbes SA, Beare D, Gunasekaran P, Leung K, Bindal N, Boutselakis H, Ding M, Bamford S, Cole C, Ward S, Kok CY, Jia M, De T, Teague JW, Stratton MR, McDermott

U, Campbell PJ (2015) COSMIC: exploring the world's knowledge of somatic mutations in human cancer. *Nucleic Acids Res* **43:** D805-811

Gentleman RC, Carey VJ, Bates DM, Bolstad B, Dettling M, Dudoit S, Ellis B, Gautier L, Ge Y, Gentry J, Hornik K, Hothorn T, Huber W, Iacus S, Irizarry R, Leisch F, Li C, Maechler M, Rossini AJ, Sawitzki G, Smith C, Smyth G, Tierney L, Yang JY, Zhang J (2004) Bioconductor: open software development for computational biology and bioinformatics. *Genome Biol* **5:** R80

George SH, Gertsenstein M, Vintersten K, Korets-Smith E, Murphy J, Stevens ME, Haigh JJ, Nagy A (2007) Developmental and adult phenotyping directly from mutant embryonic stem cells. *Proc Natl Acad Sci U S A* **104:** 4455-4460

Hobeika E, Thiemann S, Storch B, Jumaa H, Nielsen PJ, Pelanda R, Reth M (2006) Testing gene function early in the B cell lineage in mb1-cre mice. *Proc Natl Acad Sci U S A* **103:** 13789-13794

Irizarry RA, Bolstad BM, Collin F, Cope LM, Hobbs B, Speed TP (2003a) Summaries of Affymetrix GeneChip probe level data. *Nucleic Acids Res* **31:** e15

Irizarry RA, Hobbs B, Collin F, Beazer-Barclay YD, Antonellis KJ, Scherf U, Speed TP (2003b) Exploration, normalization, and summaries of high density oligonucleotide array probe level data. *Biostatistics* **4:** 249-264

Kim D, Pertea G, Trapnell C, Pimentel H, Kelley R, Salzberg SL (2013) TopHat2: accurate alignment of transcriptomes in the presence of insertions, deletions and gene fusions. *Genome Biol* **14:** R36

Konig M, Reichel M, Marschalek R, Haas OA, Strehl S (2002) A highly specific and sensitive fluorescence in situ hybridization assay for the detection of t(4;11)(q21;q23) and concurrent submicroscopic deletions in acute leukaemias. *Br J Haematol* **116:** 758-764

Kumar P, Henikoff S, Ng PC (2009) Predicting the effects of coding non-synonymous variants on protein function using the SIFT algorithm. *Nat Protoc* **4:** 1073-1081

Li H, Durbin R (2009) Fast and accurate short read alignment with Burrows-Wheeler transform. *Bioinformatics* **25:** 1754-1760

Li H, Durbin R (2010) Fast and accurate long-read alignment with Burrows-Wheeler transform. *Bioinformatics* **26:** 589-595

Li H, Handsaker B, Wysoker A, Fennell T, Ruan J, Homer N, Marth G, Abecasis G, Durbin R (2009) The Sequence Alignment/Map format and SAMtools. *Bioinformatics* **25:** 2078-2079

Love MI, Huber W, Anders S (2014) Moderated estimation of fold change and dispersion for RNA-seq data with DESeq2. *Genome Biol* **15:** 550

Mainardi S, Mijimolle N, Francoz S, Vicente-Duenas C, Sanchez-Garcia I, Barbacid M (2014) Identification of cancer initiating cells in K-Ras driven lung adenocarcinoma. *Proc Natl Acad Sci U S A* **111:** 255-260

Mammana A, and Helmuth J. (2016) bamsignals: Extract read count signals from bam files. R package version 1.8.0.

McLaren W, Pritchard B, Rios D, Chen Y, Flicek P, Cunningham F (2010) Deriving the consequences of genomic variants with the Ensembl API and SNP Effect Predictor. *Bioinformatics* **26:** 2069-2070

Miles C, Sanchez MJ, Sinclair A, Dzierzak E (1997) Expression of the Ly-6E.1 (Sca-1) transgene in adult hematopoietic stem cells and the developing mouse embryo. *Development* **124:** 537-547

Mootha VK, Lindgren CM, Eriksson KF, Subramanian A, Sihag S, Lehar J, Puigserver P, Carlsson E, Ridderstrale M, Laurila E, Houstis N, Daly MJ, Patterson N, Mesirov JP, Golub TR, Tamayo P, Spiegelman B, Lander ES, Hirschhorn JN, Altshuler D, Groop LC (2003) PGC-1alpha-responsive genes involved in oxidative phosphorylation are coordinately downregulated in human diabetes. *Nat Genet* **34:** 267-273

Ng OH, Erbilgin Y, Firtina S, Celkan T, Karakas Z, Aydogan G, Turkkan E, Yildirmak Y, Timur C, Zengin E, van Dongen JJ, Staal FJ, Ozbek U, Sayitoglu M (2014) Deregulated WNT signaling in childhood T-cell acute lymphoblastic leukemia. *Blood Cancer J* **4:** e192

Schebesta A, McManus S, Salvagiotto G, Delogu A, Busslinger GA, Busslinger M (2007) Transcription factor Pax5 activates the chromatin of key genes involved in B cell signaling, adhesion, migration, and immune function. *Immunity* **27:** 49-63

Smedley D, Haider S, Durinck S, Pandini L, Provero P, Allen J, Arnaiz O, Awedh MH, Baldock R, Barbiera G, Bardou P, Beck T, Blake A, Bonierbale M, Brookes AJ, Bucci G, Buetti I, Burge S, Cabau C, Carlson JW, Chelala C, Chrysostomou C, Cittaro D, Collin O, Cordova R, Cutts RJ, Dassi E, Di Genova A, Djari A, Esposito A, Estrella H, Eyras E, Fernandez-Banet J, Forbes S, Free RC, Fujisawa T, Gadaleta E, Garcia-Manteiga JM, Goodstein D, Gray K, Guerra-Assuncao JA, Haggarty B, Han DJ, Han BW, Harris T, Harshbarger J, Hastings RK, Hayes RD, Hoede C, Hu S, Hu ZL, Hutchins L, Kan Z, Kawaji H, Keliet A, Kerhornou A, Kim S, Kinsella R, Klopp C, Kong L, Lawson D, Lazarevic D, Lee JH, Letellier T, Li CY, Lio P, Liu CJ, Luo J, Maass A, Mariette J, Maurel T, Merella S, Mohamed AM, Moreews F, Nabihoudine I, Ndegwa N, Noirot C, Perez-Llamas C, Primig M, Quattrone A, Quesneville H, Rambaldi D, Reecy J, Riba M, Rosanoff S, Saddiq AA, Salas E, Sallou O, Shepherd R, Simon R, Sperling L, Spooner W, Staines DM, Steinbach D, Stone K, Stupka E, Teague JW, Dayem Ullah AZ, Wang J, Ware D, Wong-Erasmus M, Youens-Clark K, Zadissa A, Zhang SJ, Kasprzyk A (2015) The BioMart community portal: an innovative alternative to large, centralized data repositories. *Nucleic Acids Res* **43:** W589-598

Subramanian A, Tamayo P, Mootha VK, Mukherjee S, Ebert BL, Gillette MA, Paulovich A, Pomeroy SL, Golub TR, Lander ES, Mesirov JP (2005) Gene set enrichment analysis: a knowledge-based approach for interpreting genome-wide expression profiles. *Proc Natl Acad Sci U S A* **102:** 15545-15550

Tusher VG, Tibshirani R, Chu G (2001) Significance analysis of microarrays applied to the ionizing radiation response. *Proc Natl Acad Sci U S A* **98:** 5116-5121

Warnes GR, Bolker B, Bonebakker L, Gentleman R, Liaw WHA, Lumley T, Maechler M, Magnusson A, Moeller S, Schwartz M, Venables B. (2016) gplots: Various R Programming Tools for Plotting Data. R package version 3.0.1.

Appendix Figure S1

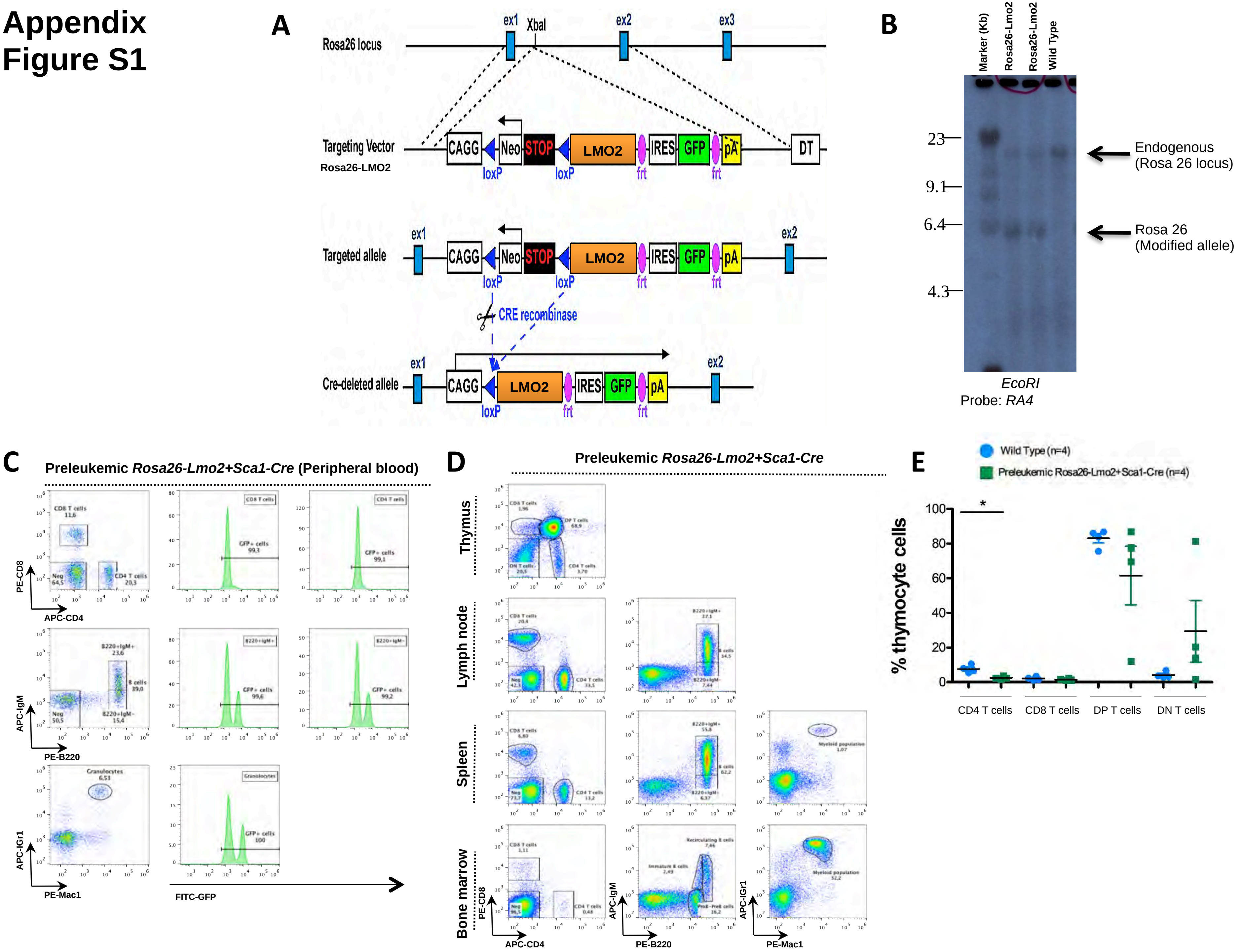

Appendix Figure S2

A

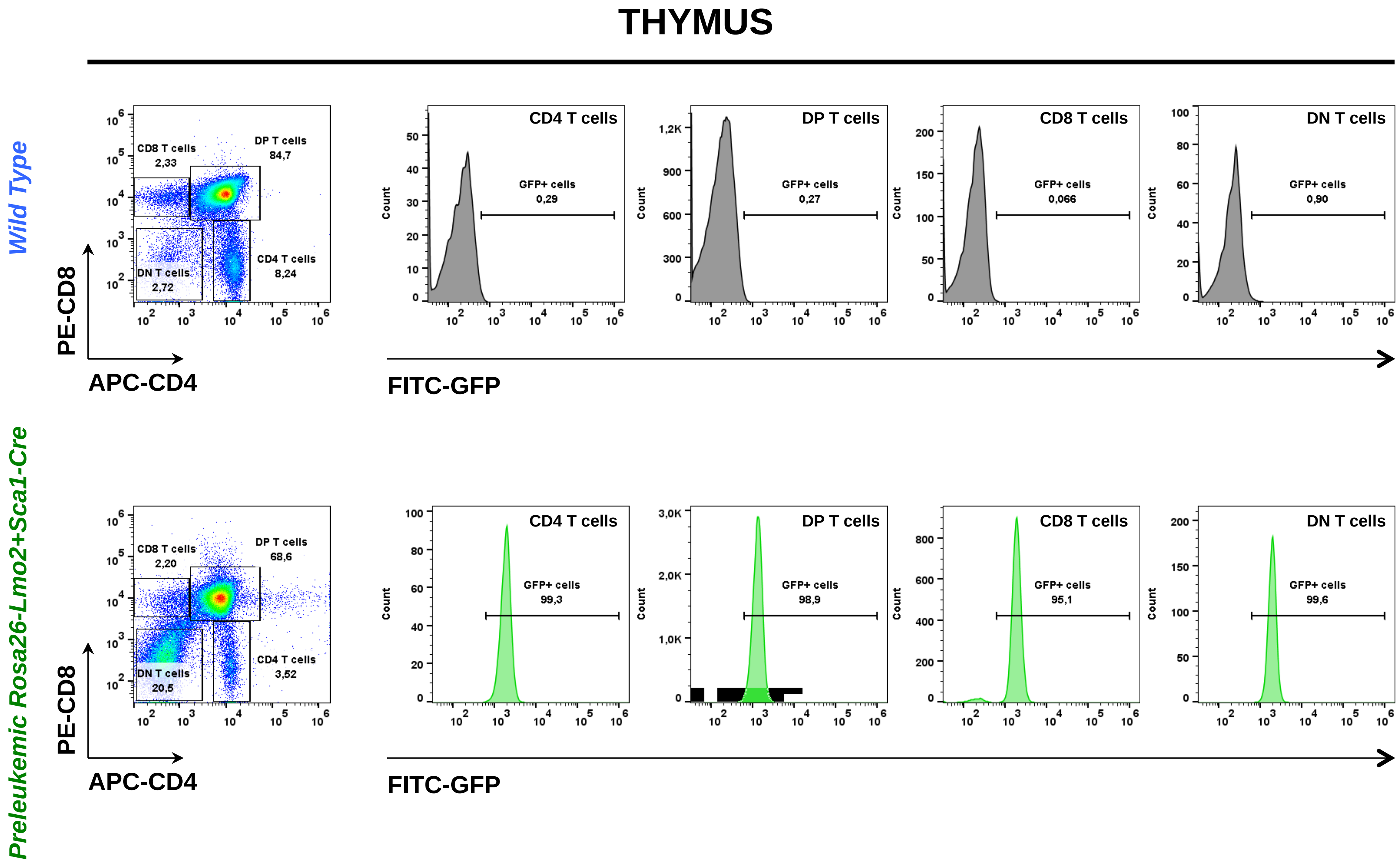

Appendix Figure S2

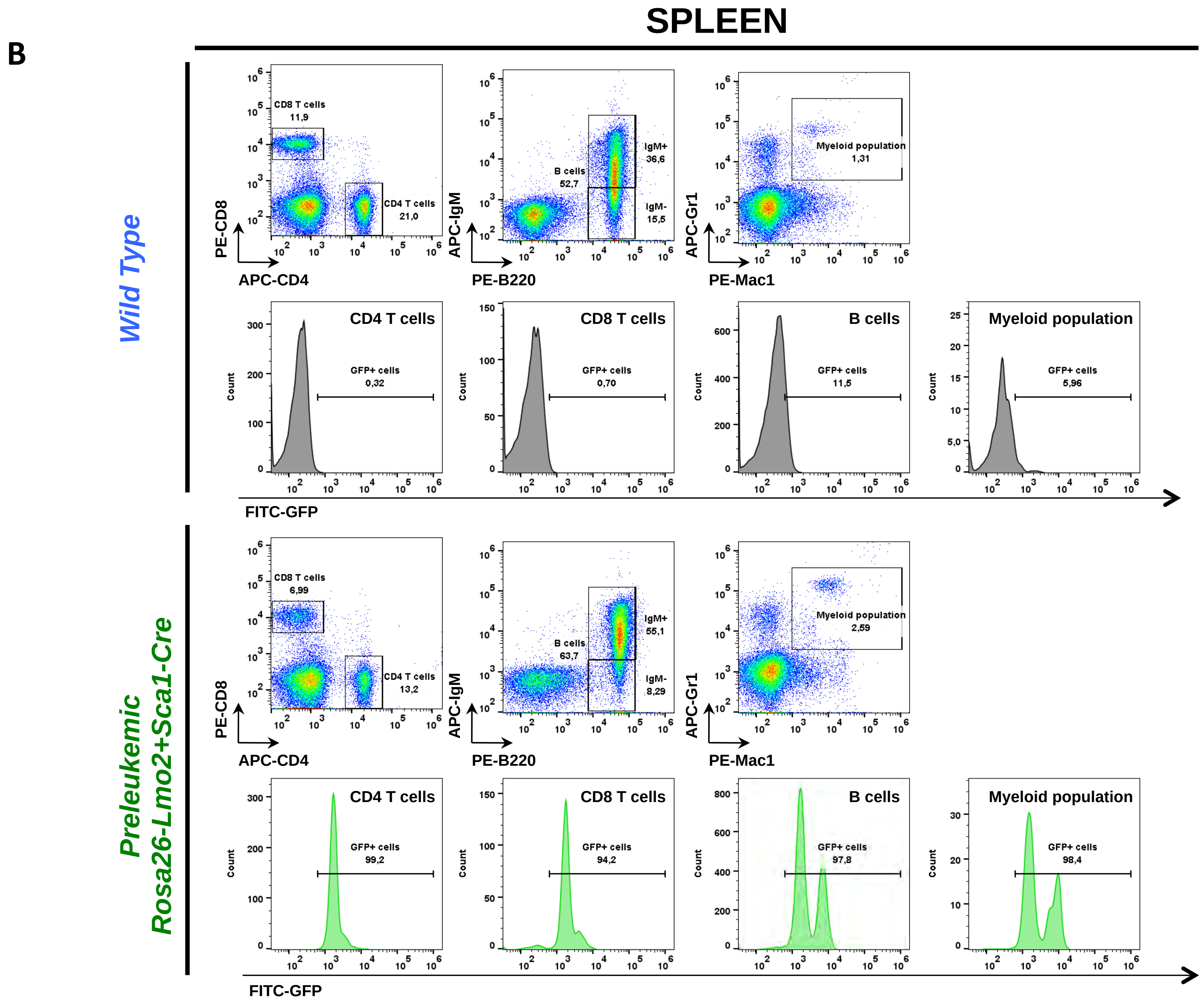

Appendix Figure S2

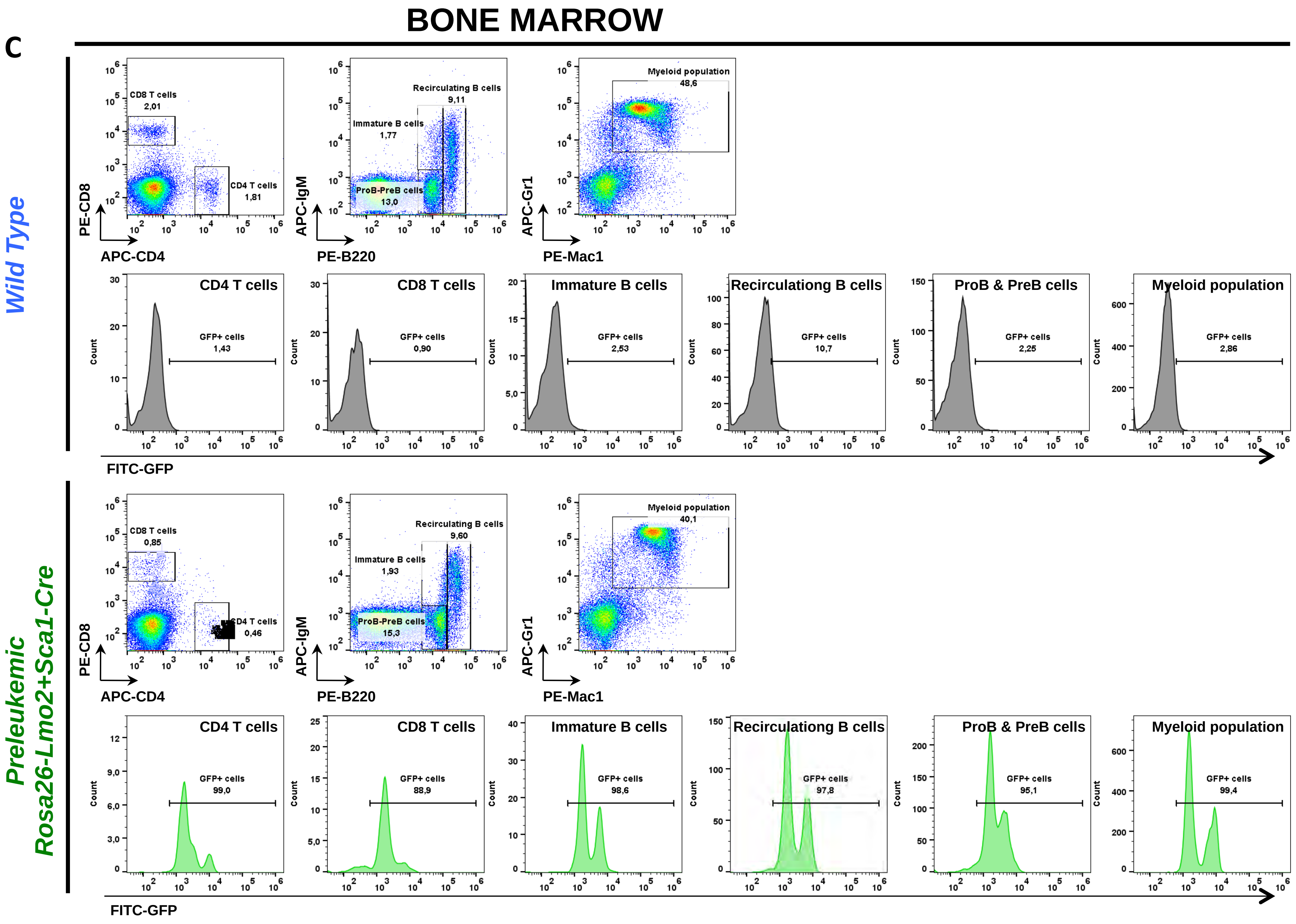

Appendix Figure S2

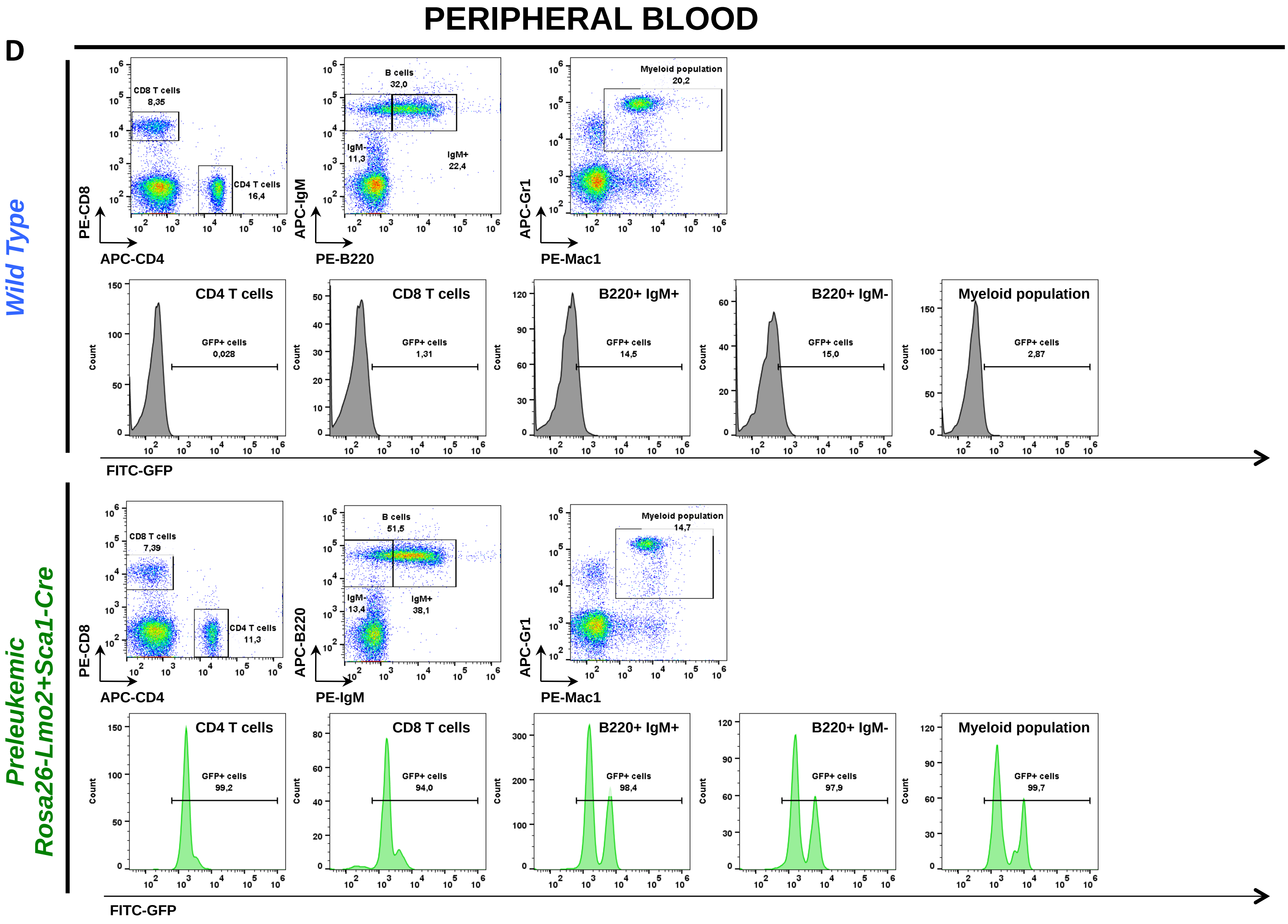

Appendix Figure S3

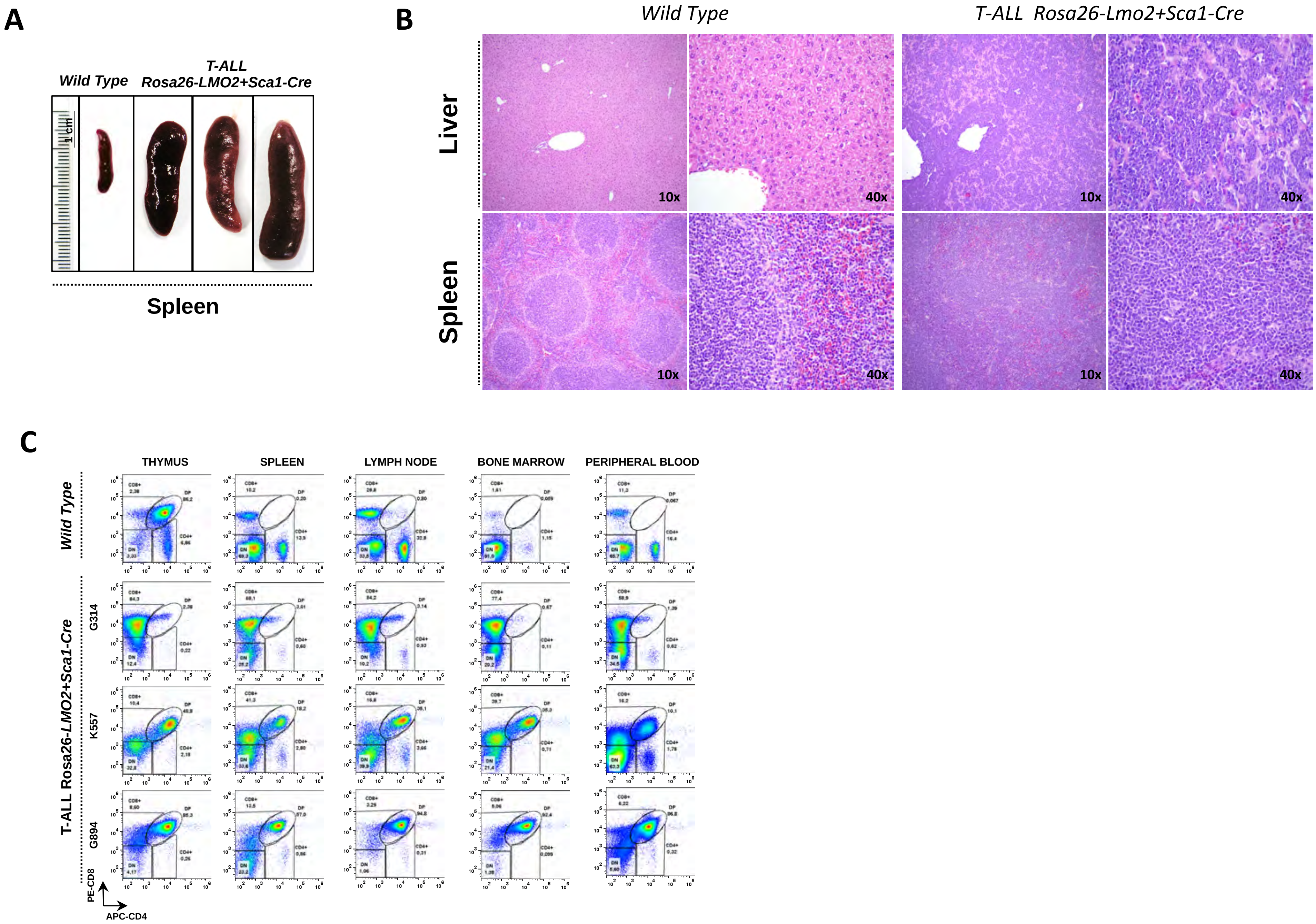

Appendix Figure S4

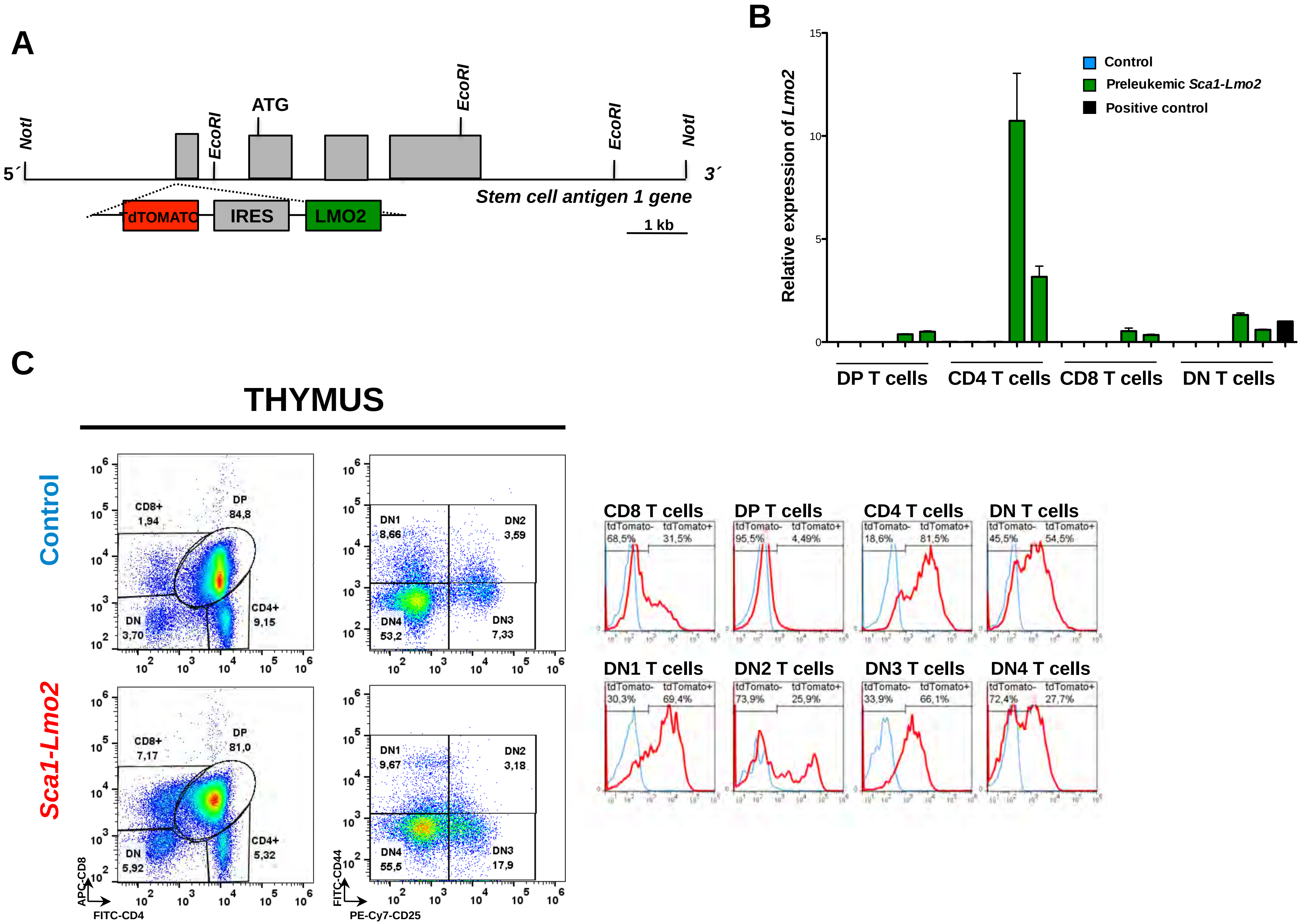

Appendix Figure S4

D

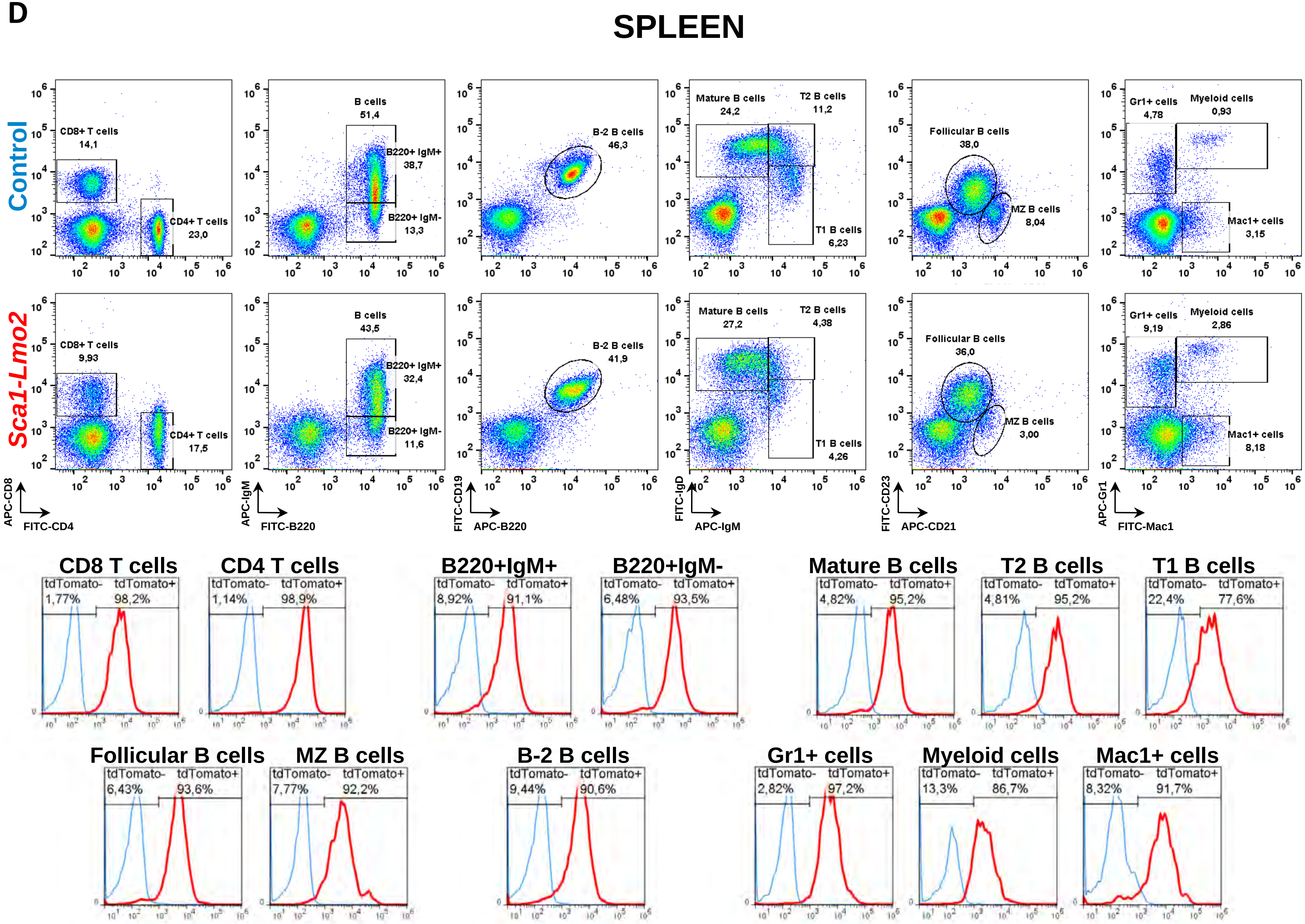

Appendix Figure S4

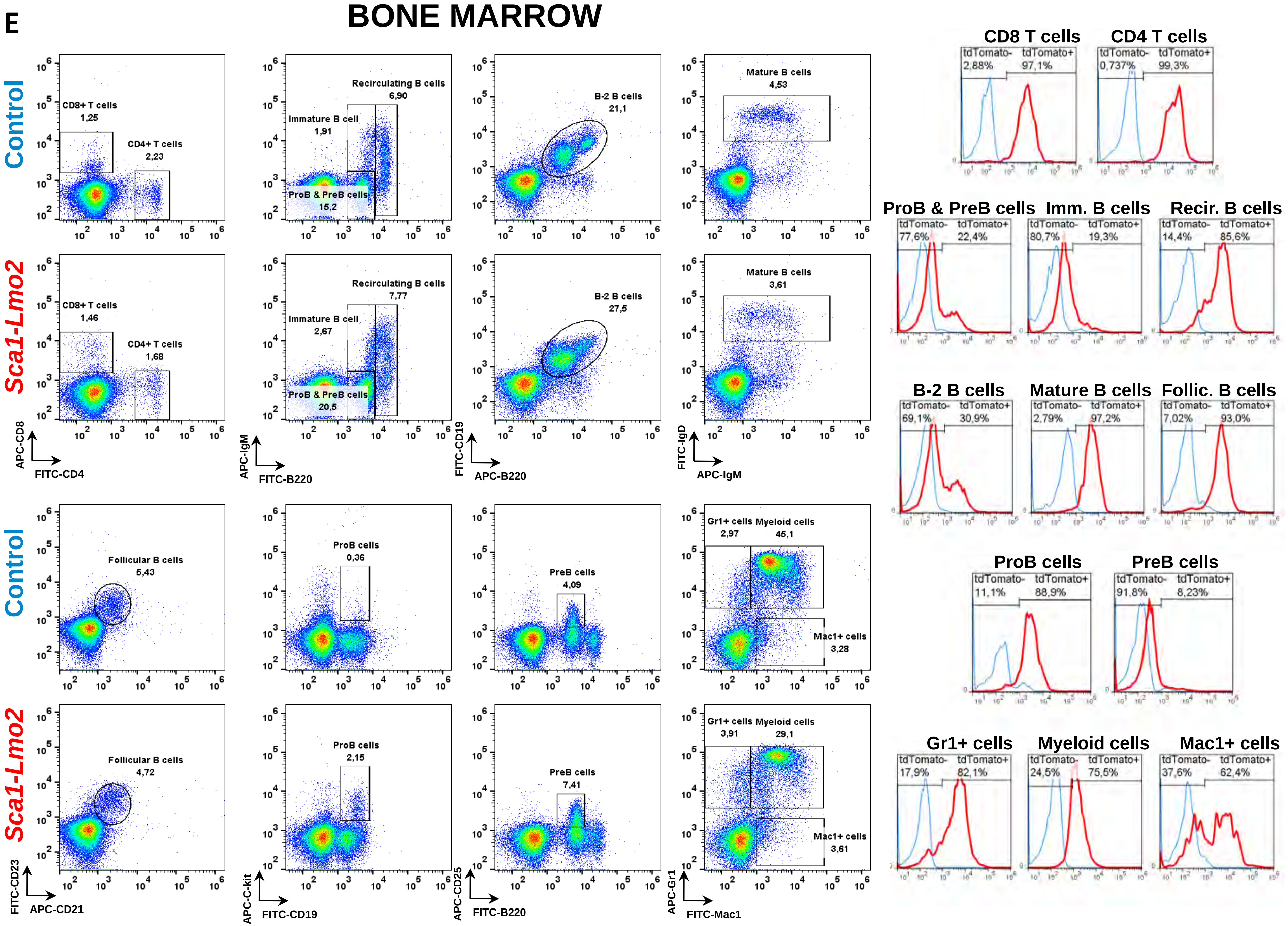

Appendix Figure S4

F

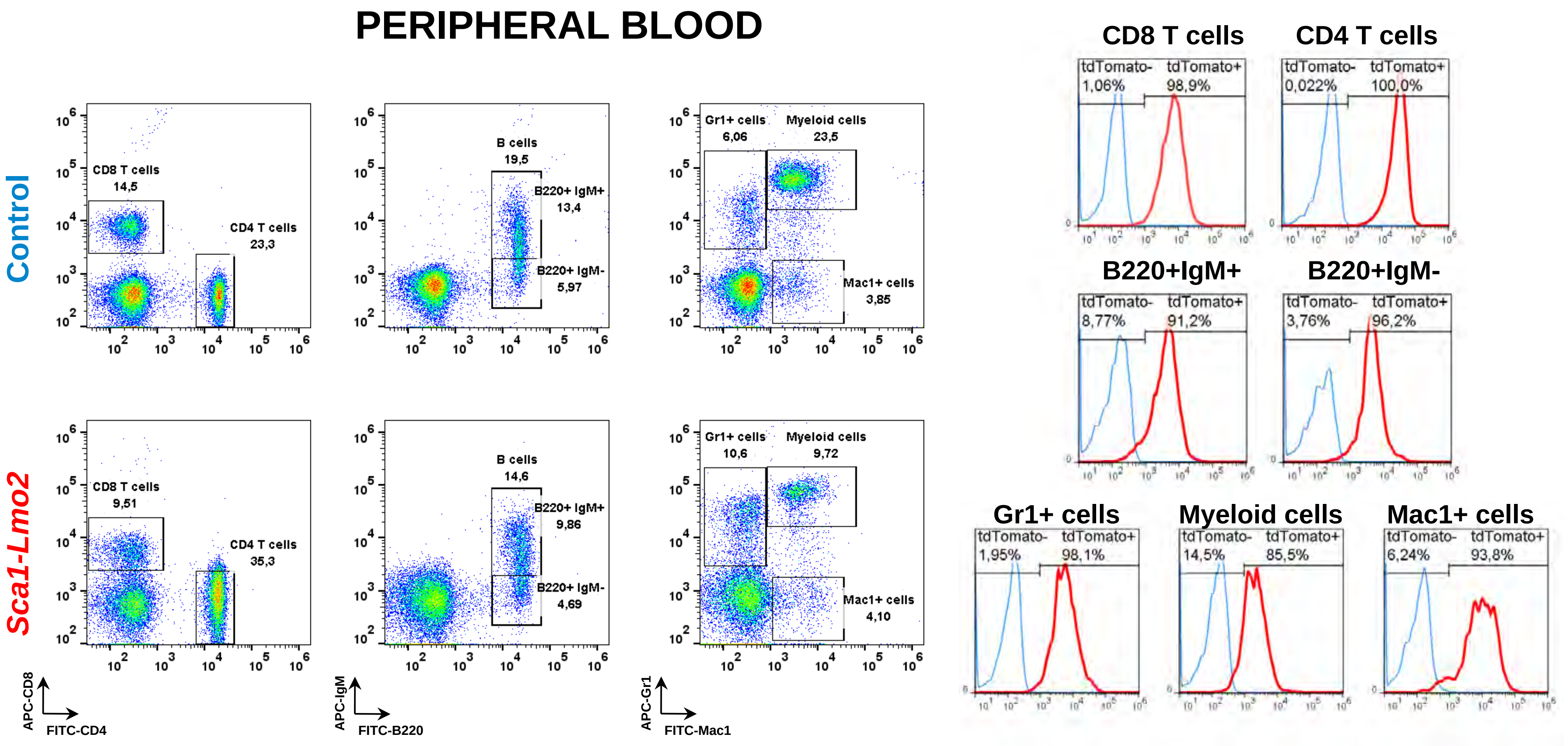

Appendix Figure S4

G

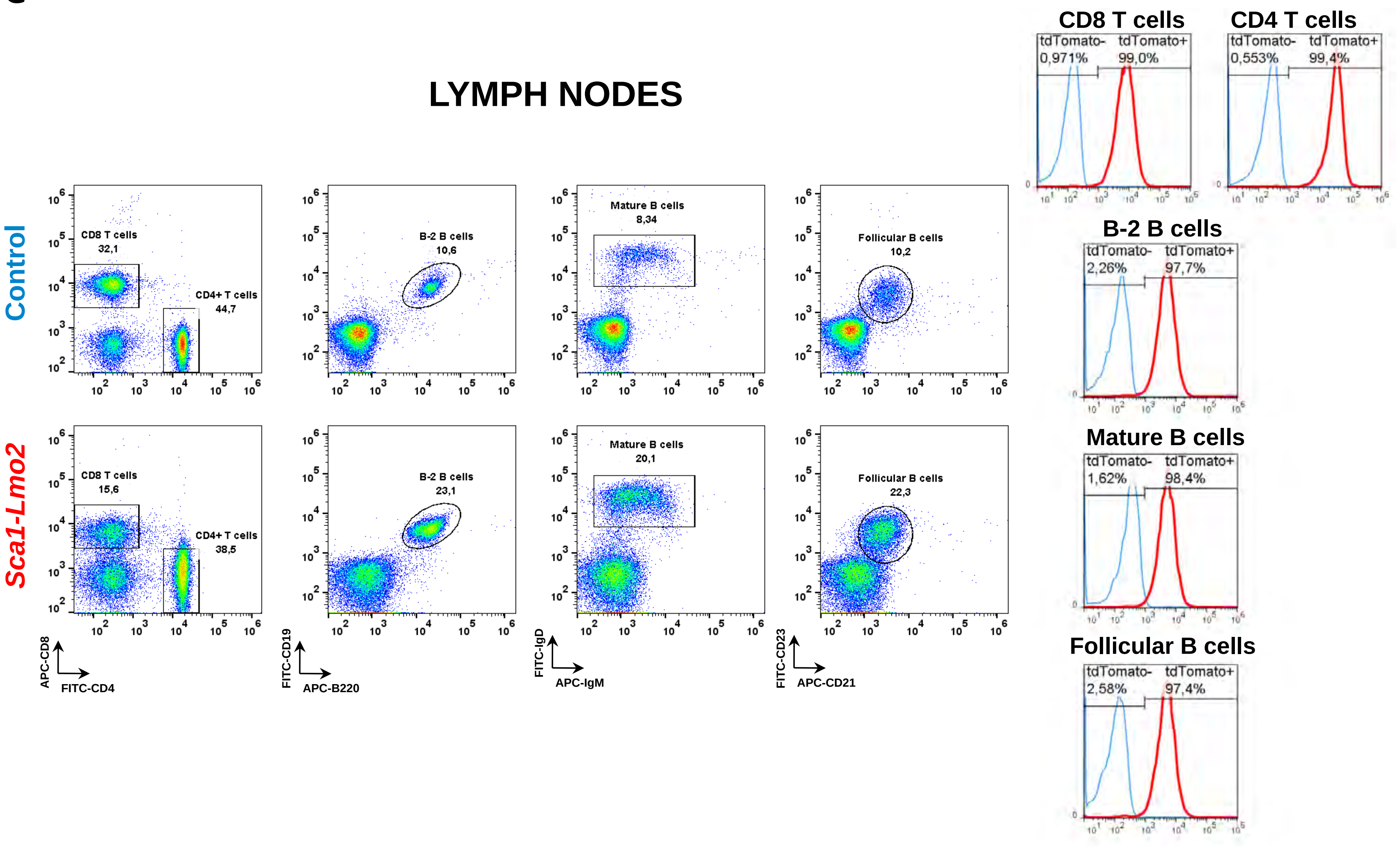

Appendix Figure S4

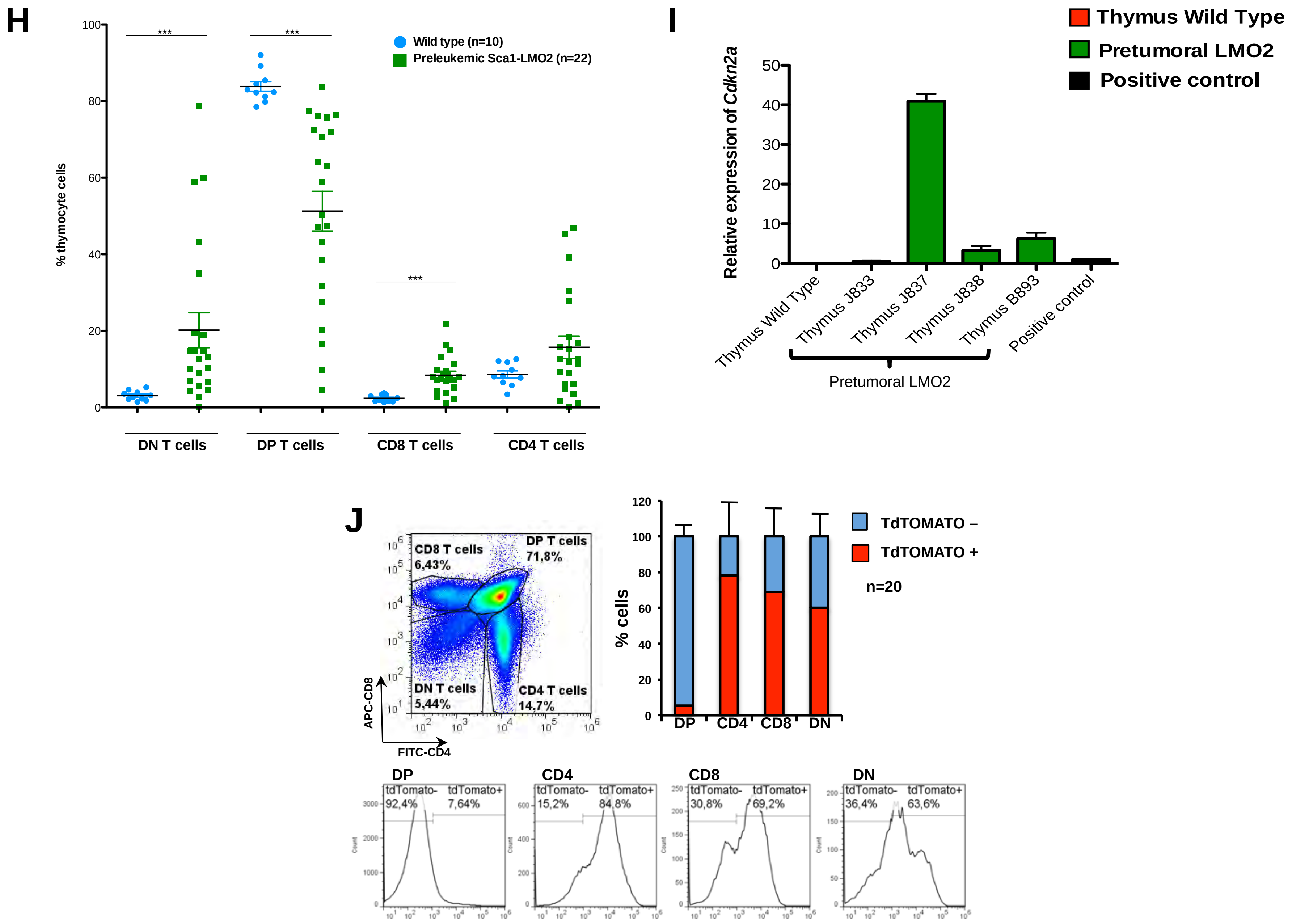

Appendix Figure S5

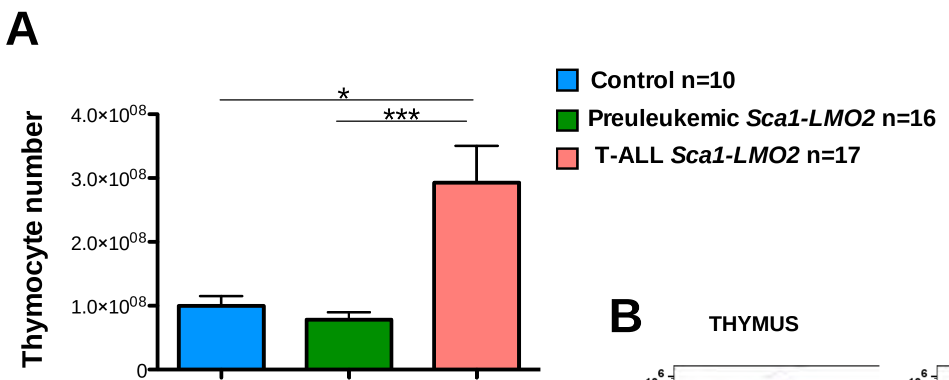

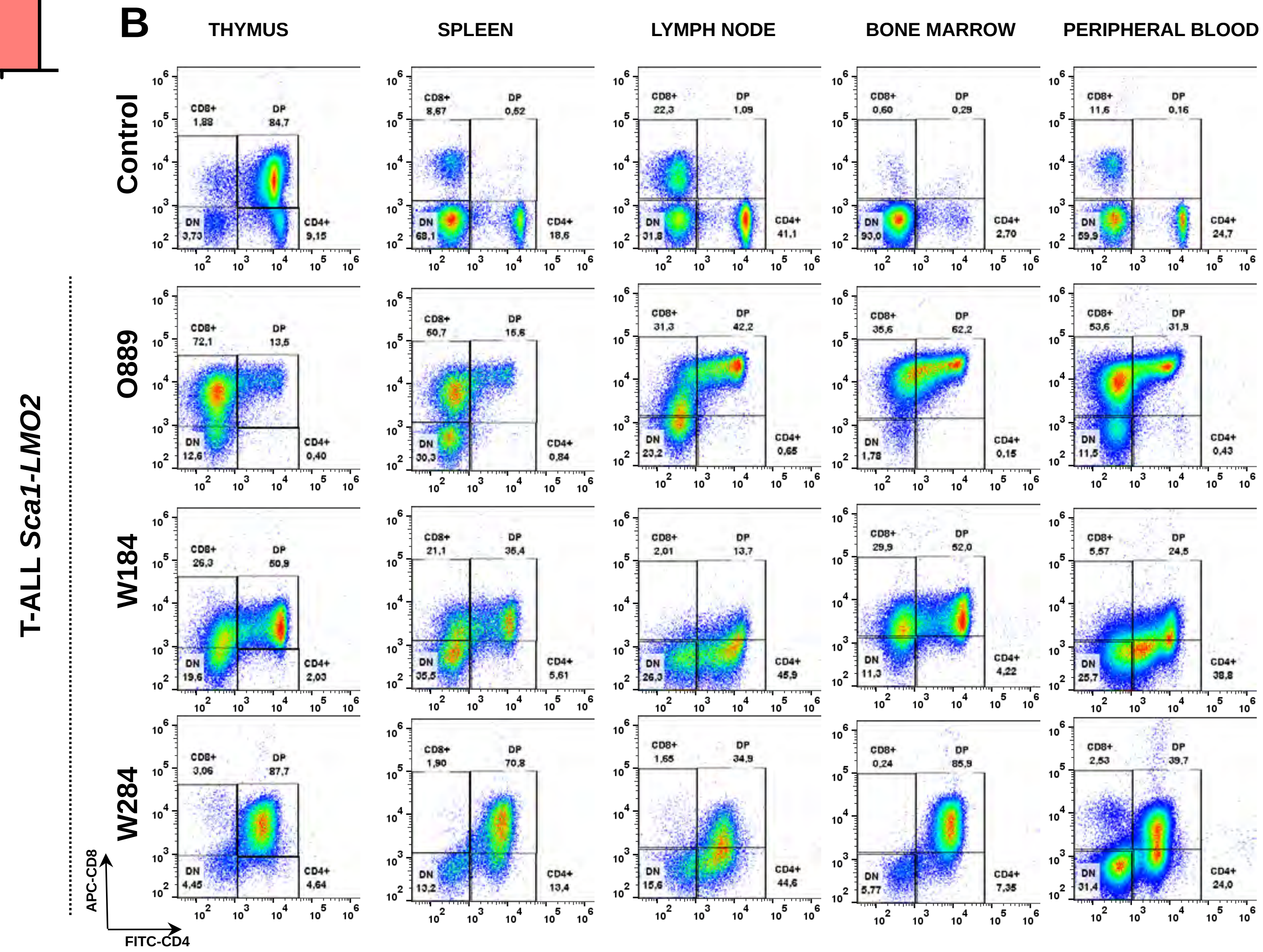

Appendix Figure S5

C

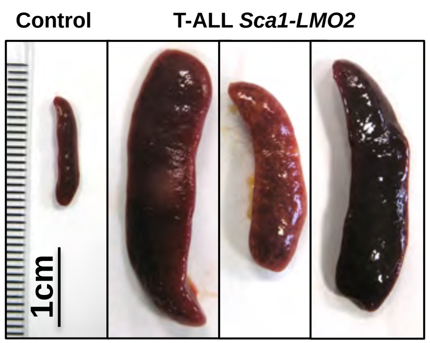

D

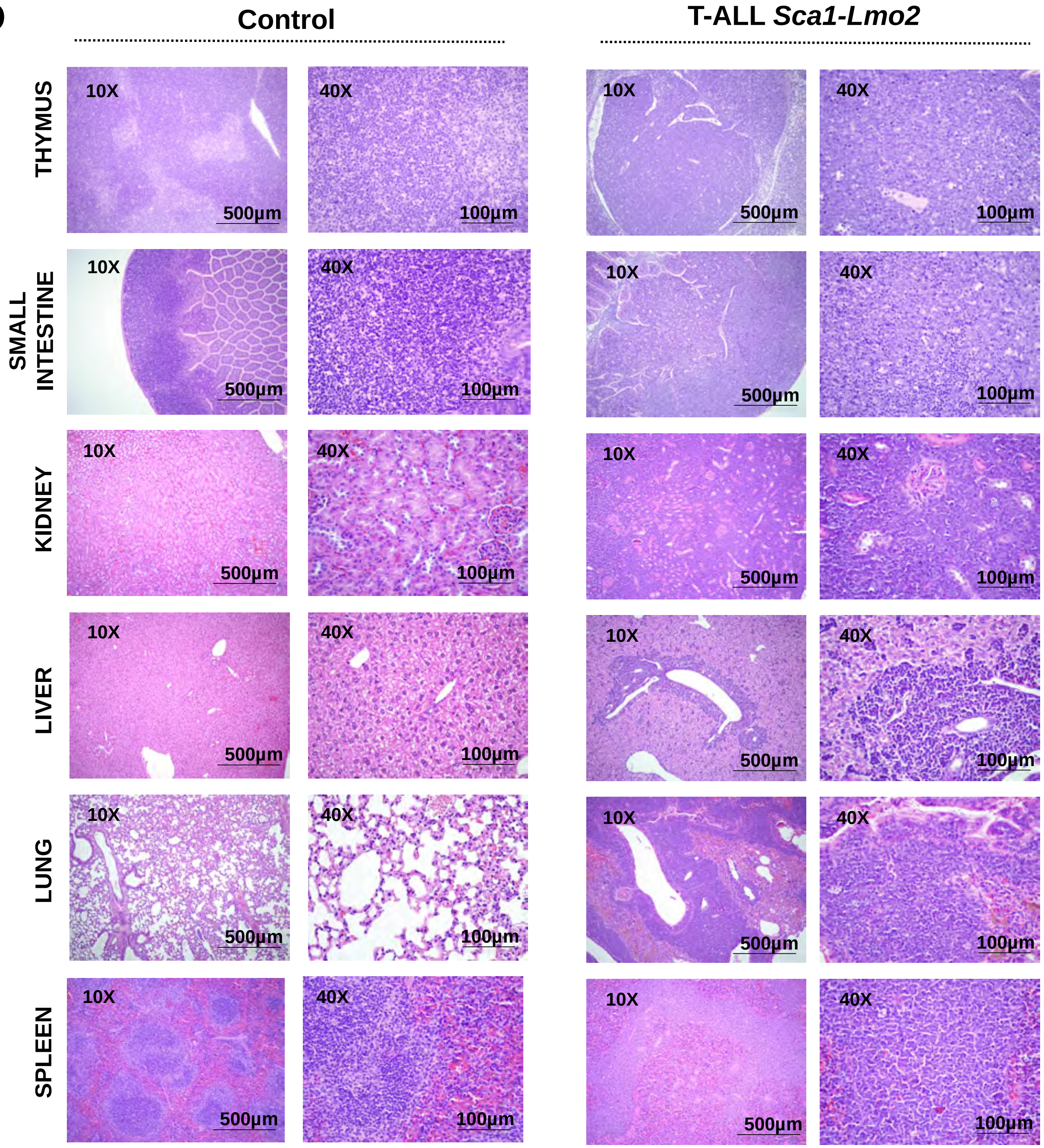

Appendix Figure S5

E

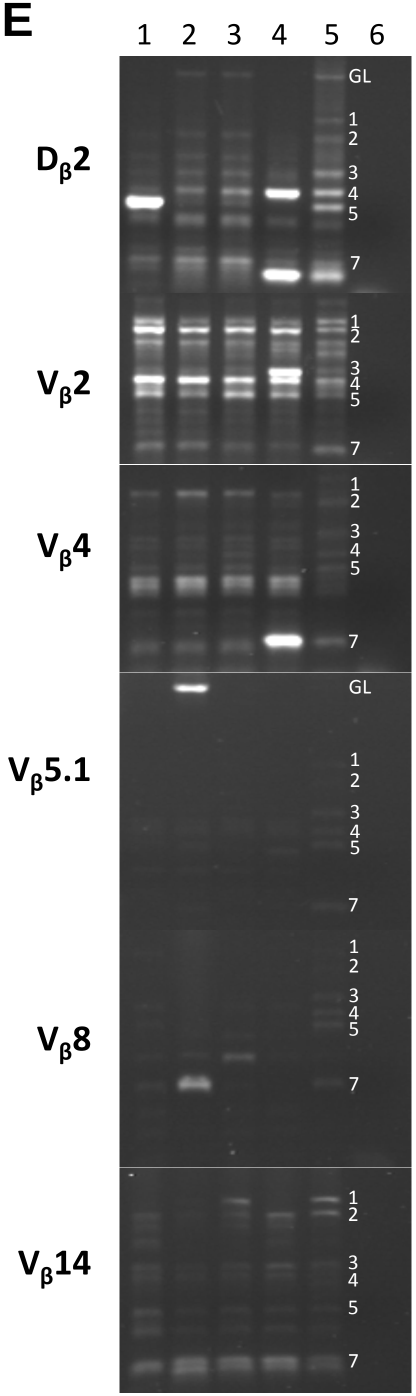

1. B892
2. B993
3. W184
4. W281
5. Double Positive T cells (WT)
6. MPW

(1–4: *Sca1-Lmo2*)

Appendix Figure S6

A

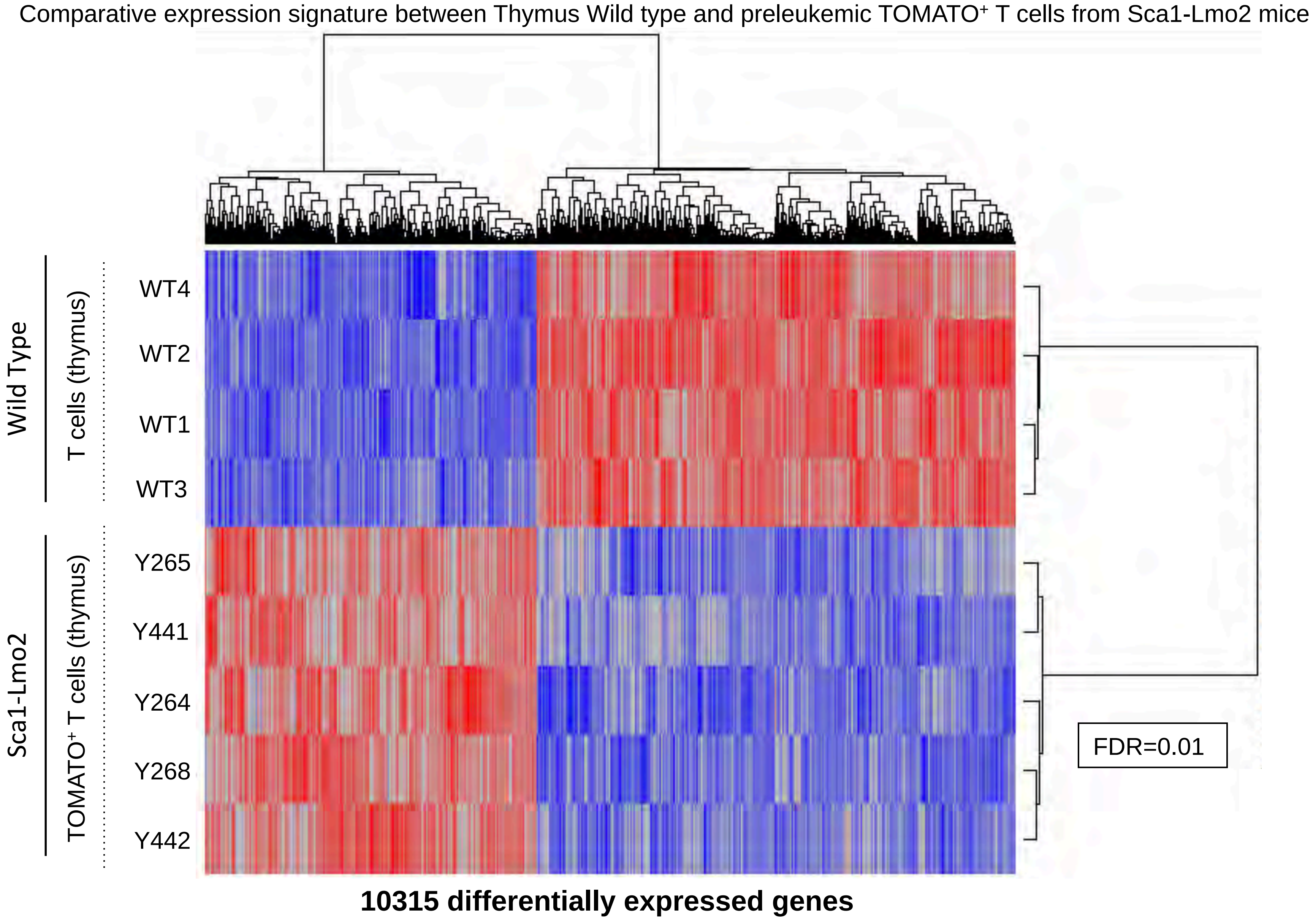

Appendix Figure S6

B

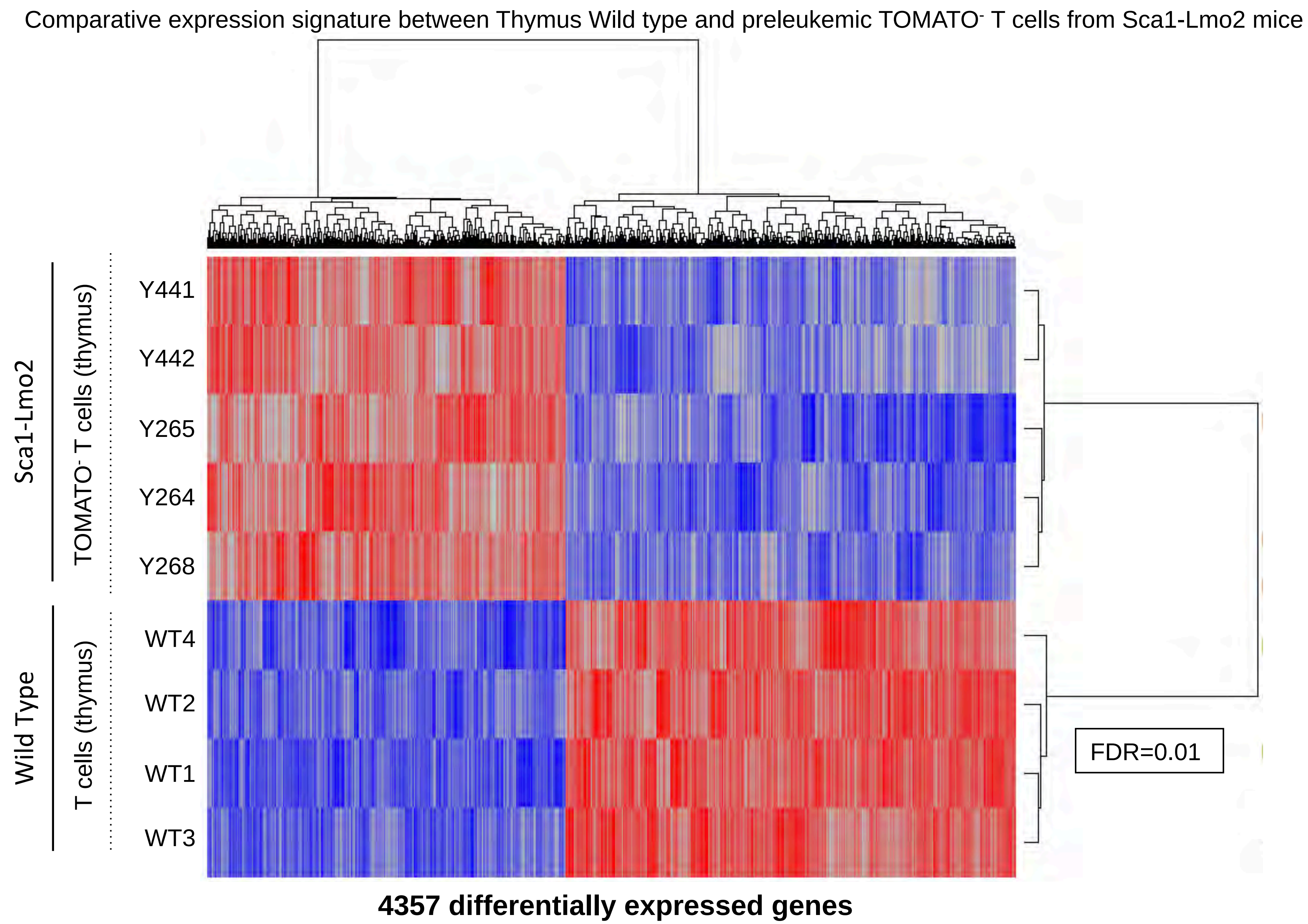

Appendix Figure S6
C

Comparative expression signature between Preleukemic TOMATO^{+} T cells and preleukemic TOMATO^{-} T cells from Sca1-Lmo2 mice

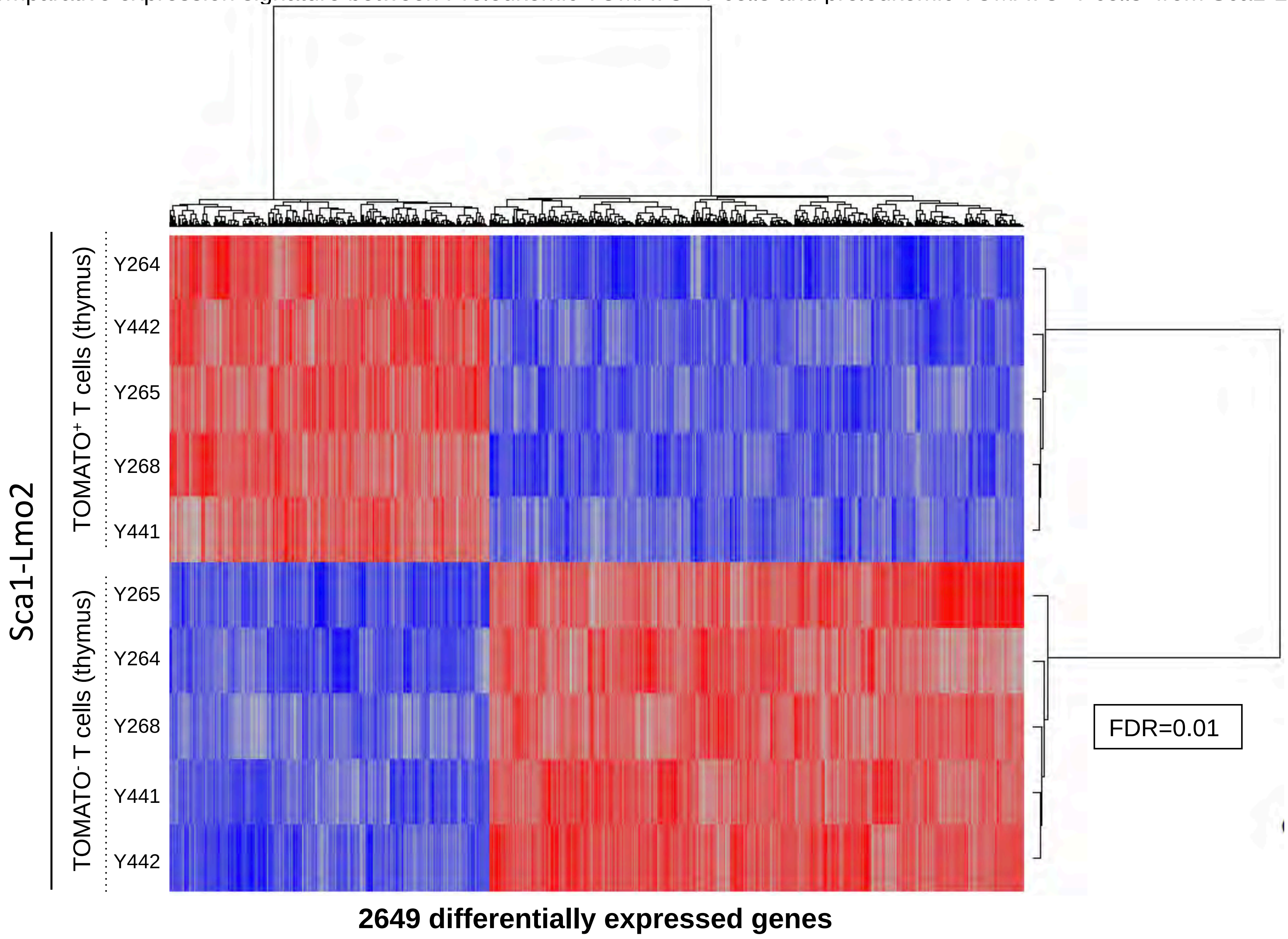

Appendix Figure S6

D

Comparative expression signature between preleukemic TOMATO^{+} T cells and leukemic T cells from Sca1-Lmo2 mice

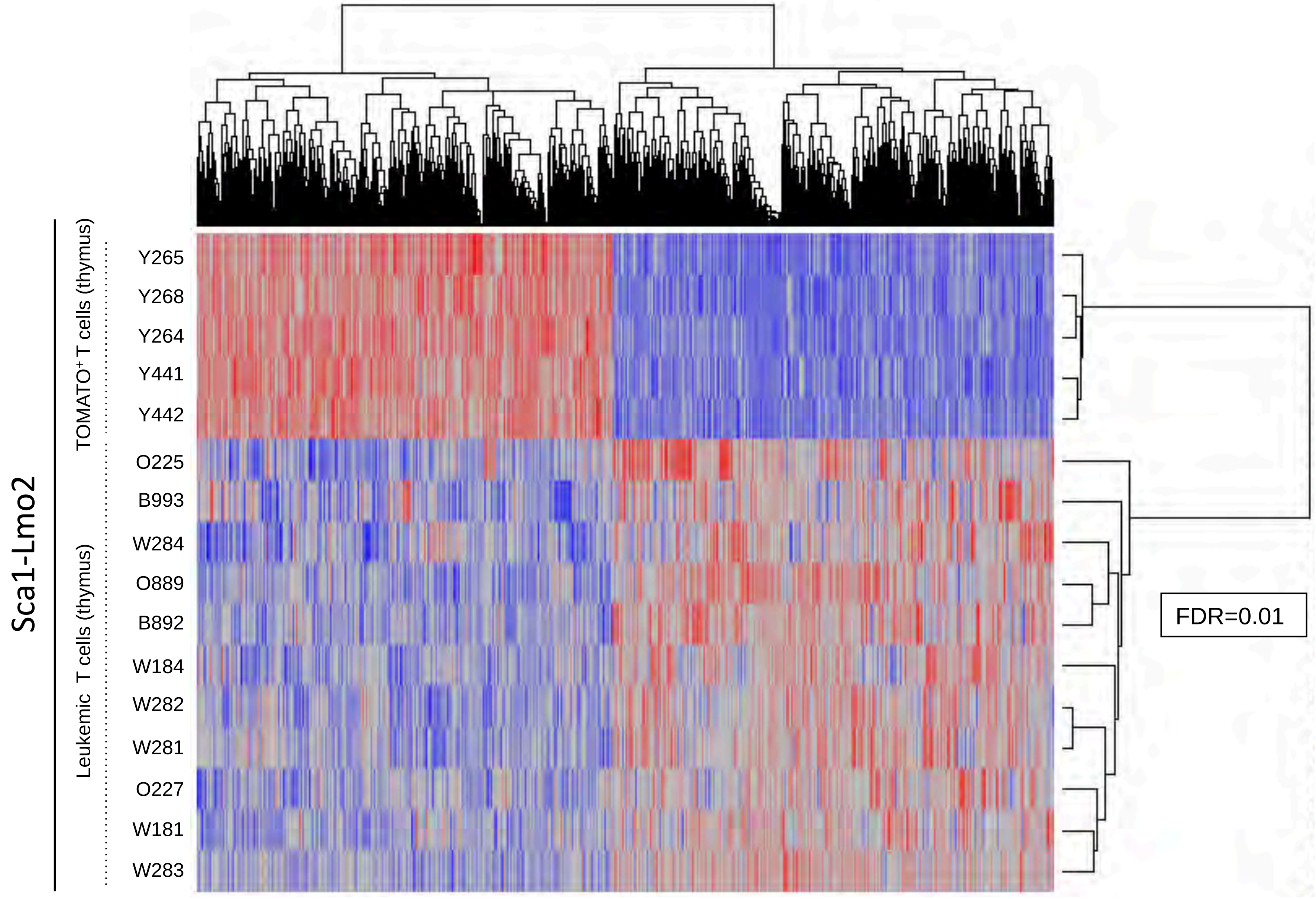

13116 differentially expressed genes

Appendix Figure S6

E

Comparative expression signature between Preleukemic TOMATO$^-$ T cells and Leukemic T cells from Sca1-Lmo2 mice

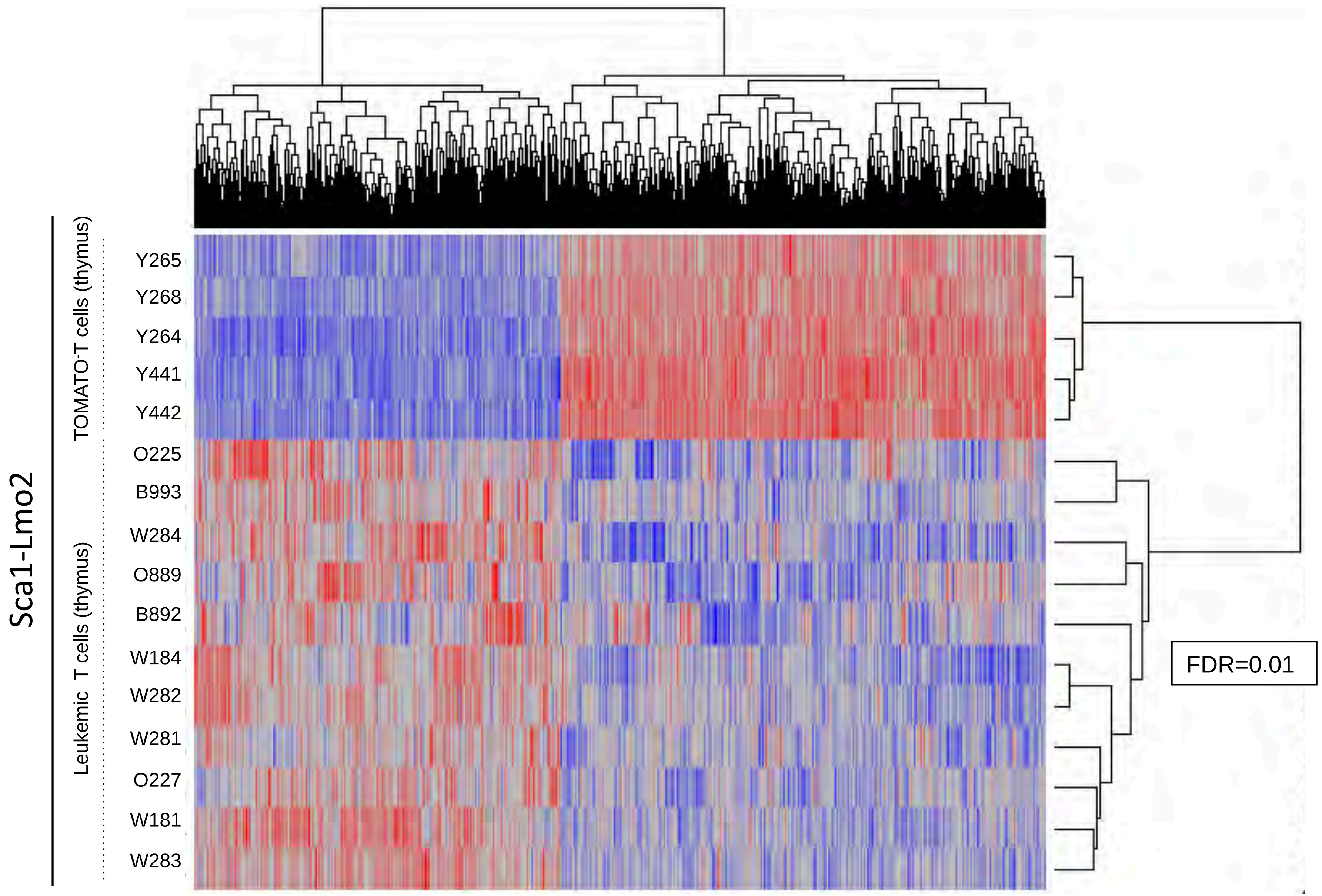

9383 differentially expressed genes

Appendix Figure S7

FISH-Human t(11;14) (p13;q11)

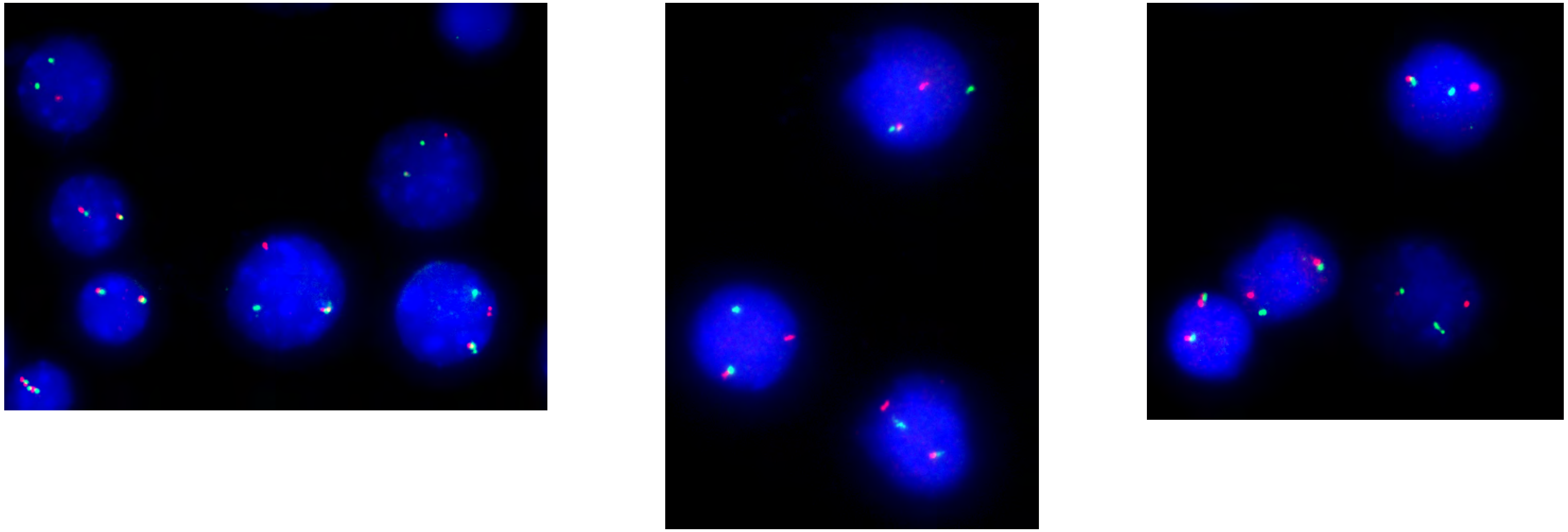

Patient-1 **Patient-2** **Patient-3**

Appendix Figure S8

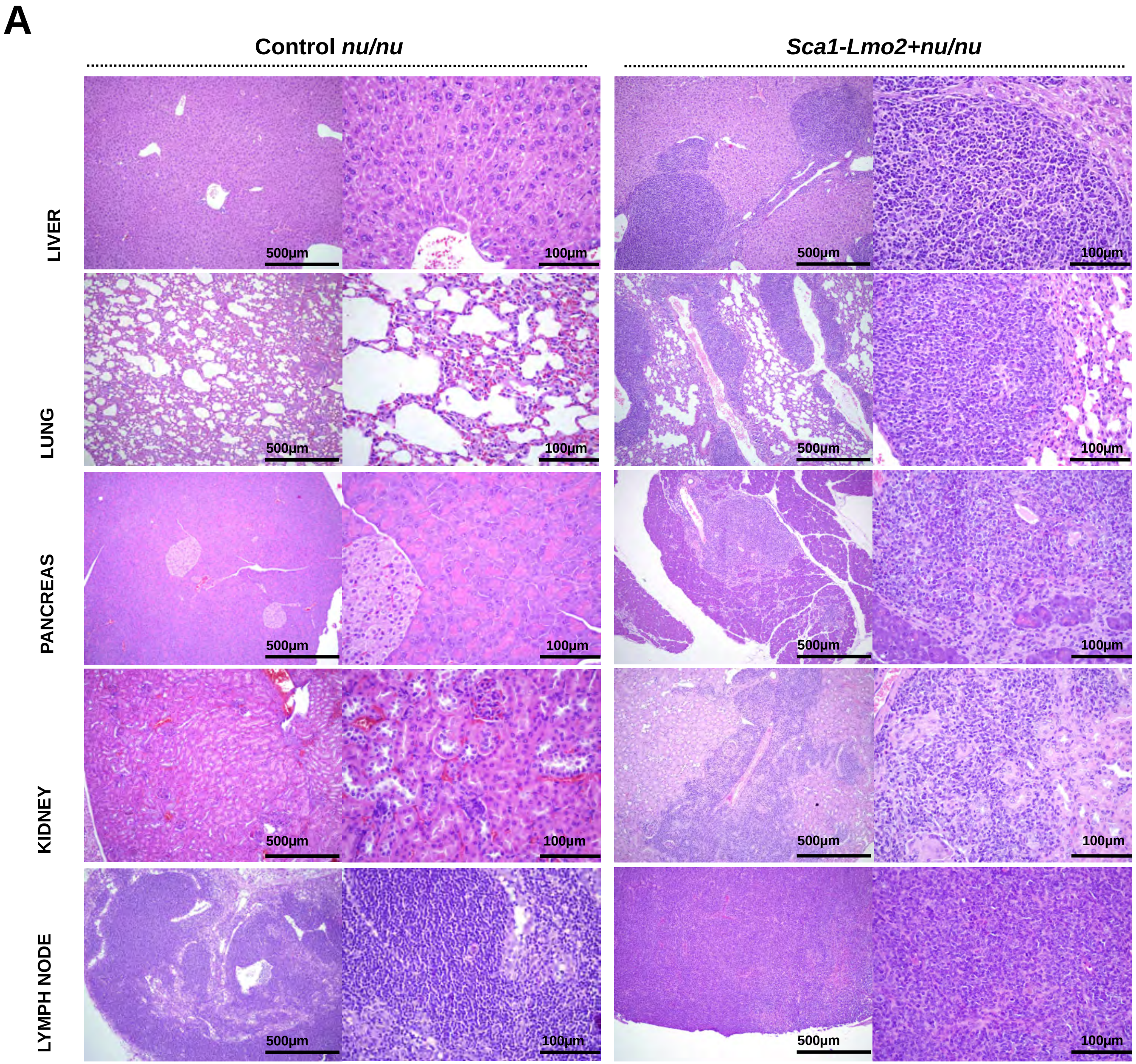

Appendix Figure S8

B

Comparative expression signature between bone marrow from control nude mice and leukemic cells from Sca1-Lmo2+nu/nu mice

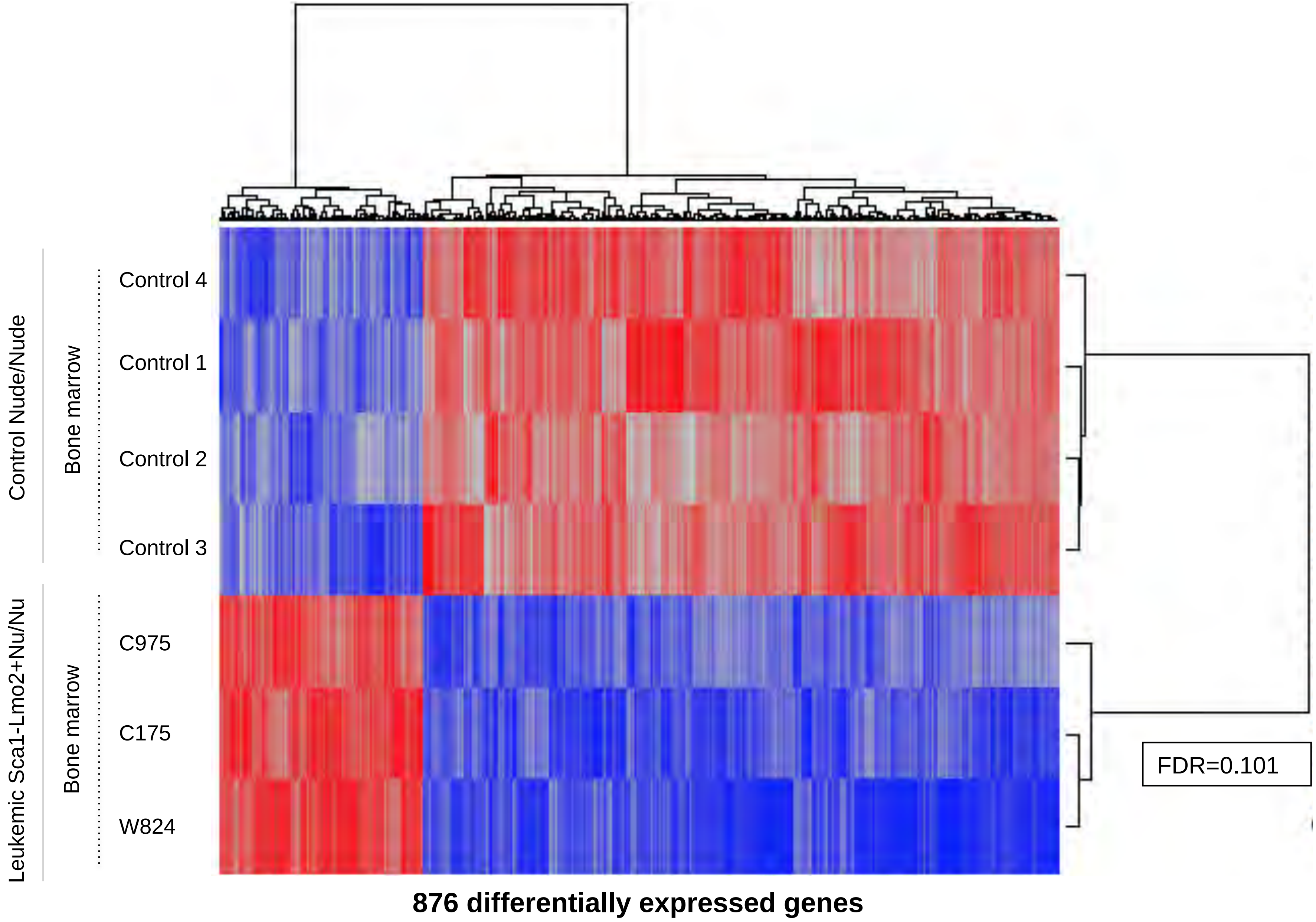

Appendix Figure S8

C

Comparative expression signature between leukemic cells from Sca1-Lmo2+nu/nu mice and leukemic T cells from Sca1-Lmo2 mice

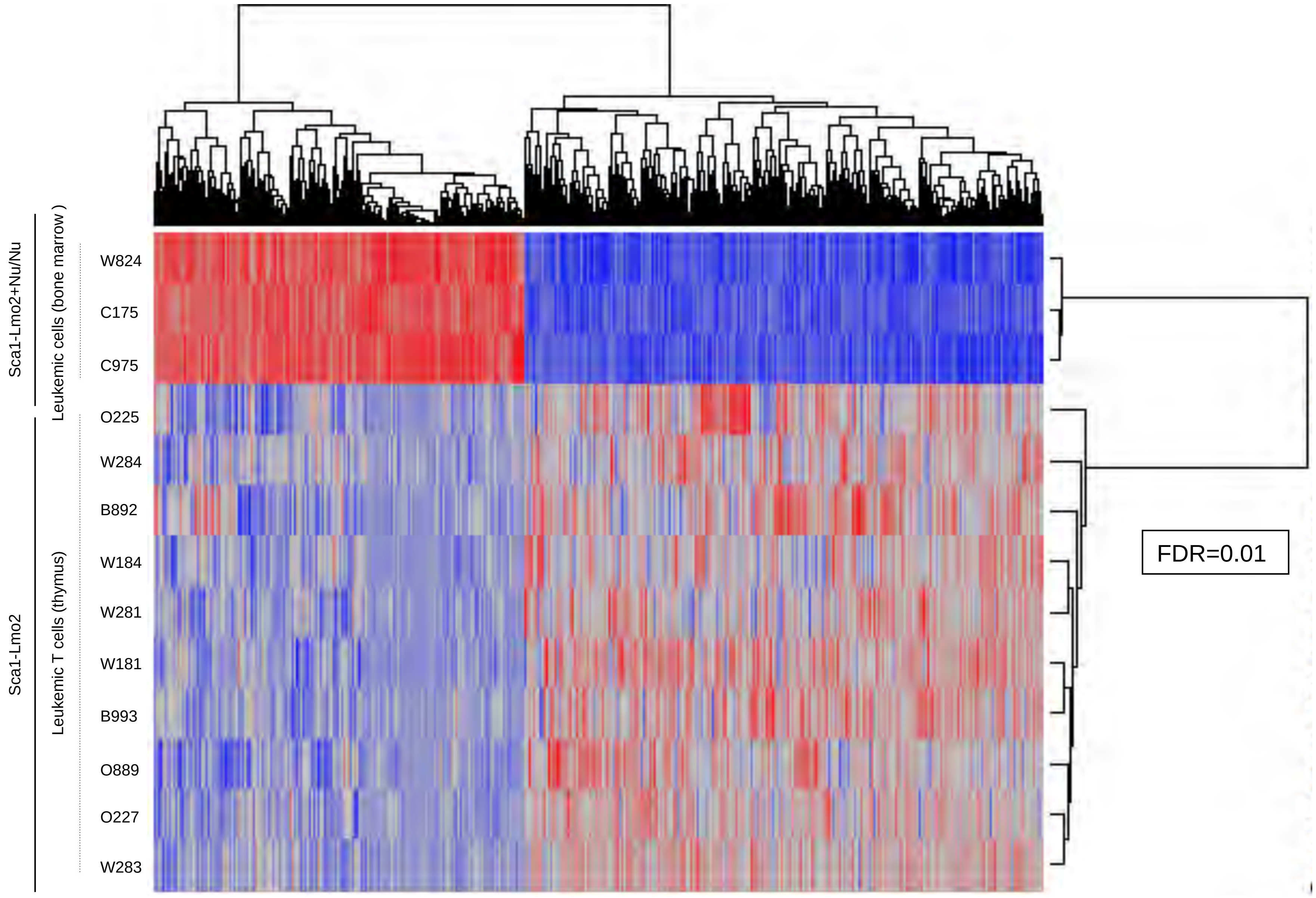

5801 differentially expressed genes

Appendix Figure S9

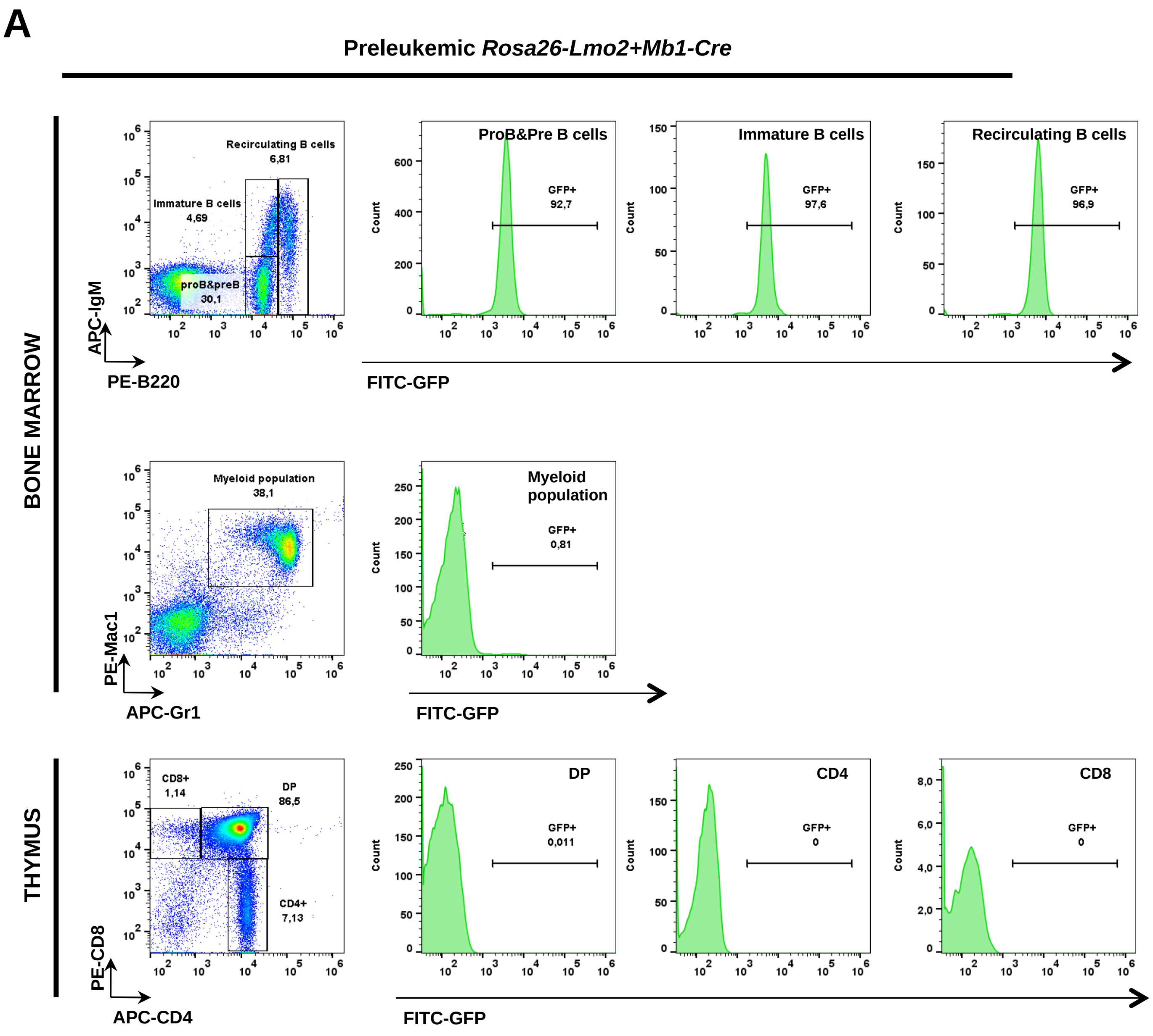

Appendix Figure S9

B

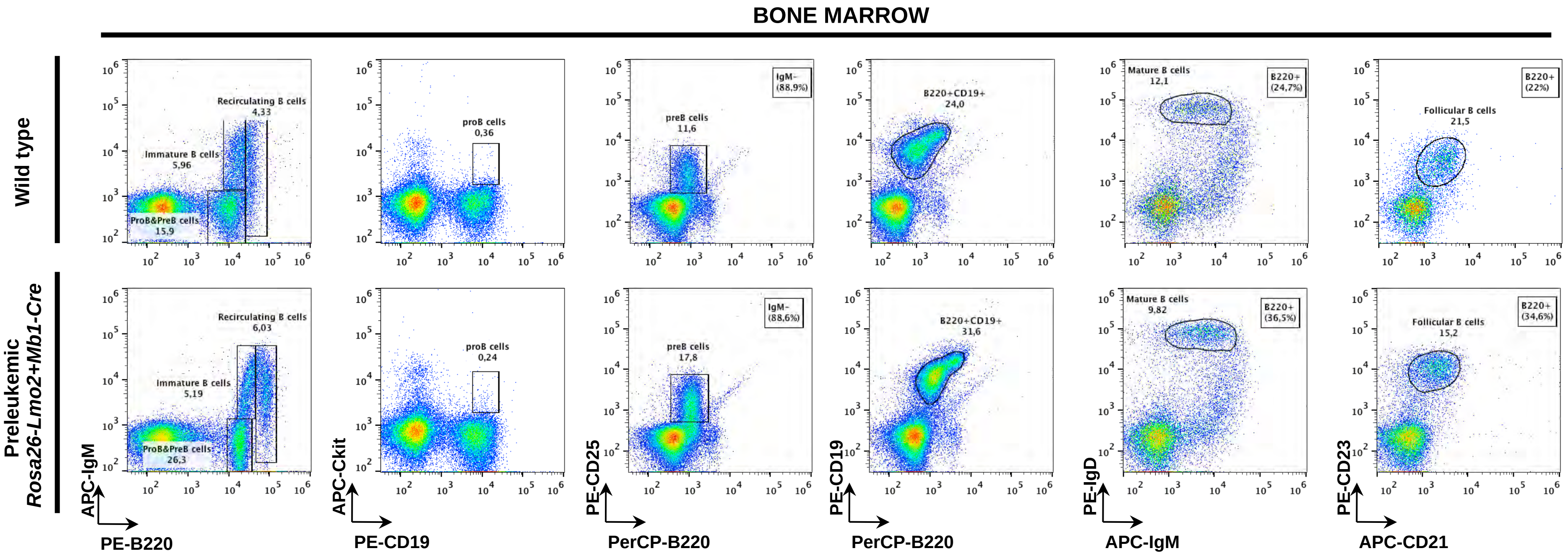

Appendix Figure S9

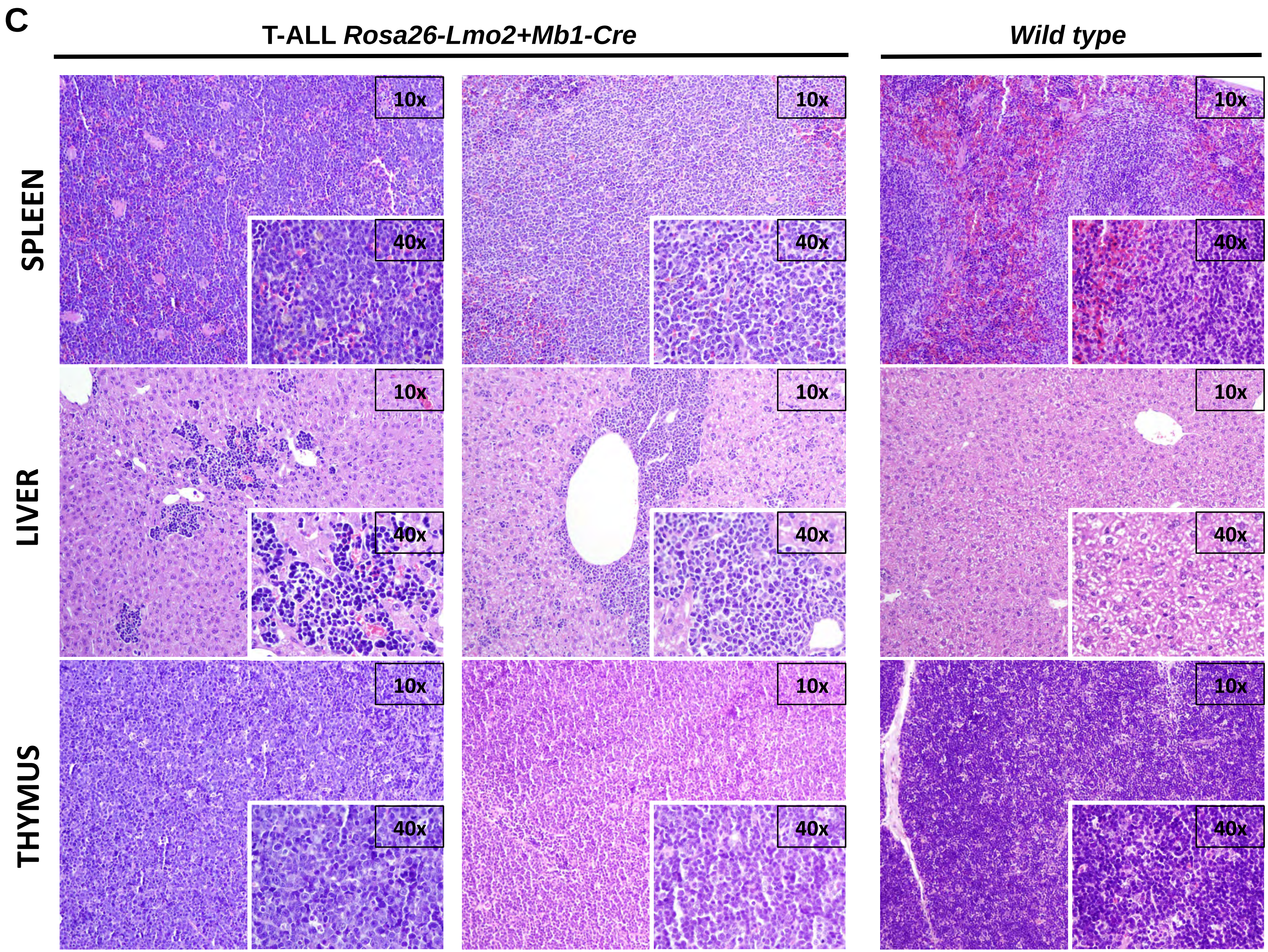

Appendix Figure S9

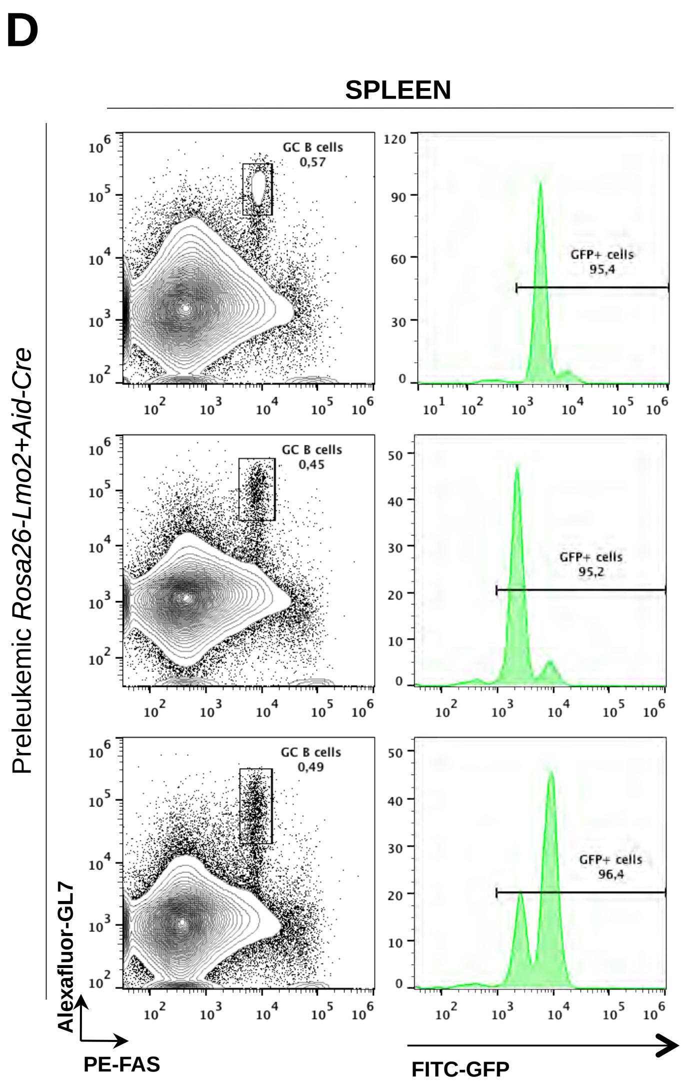

Appendix Figure S9

E

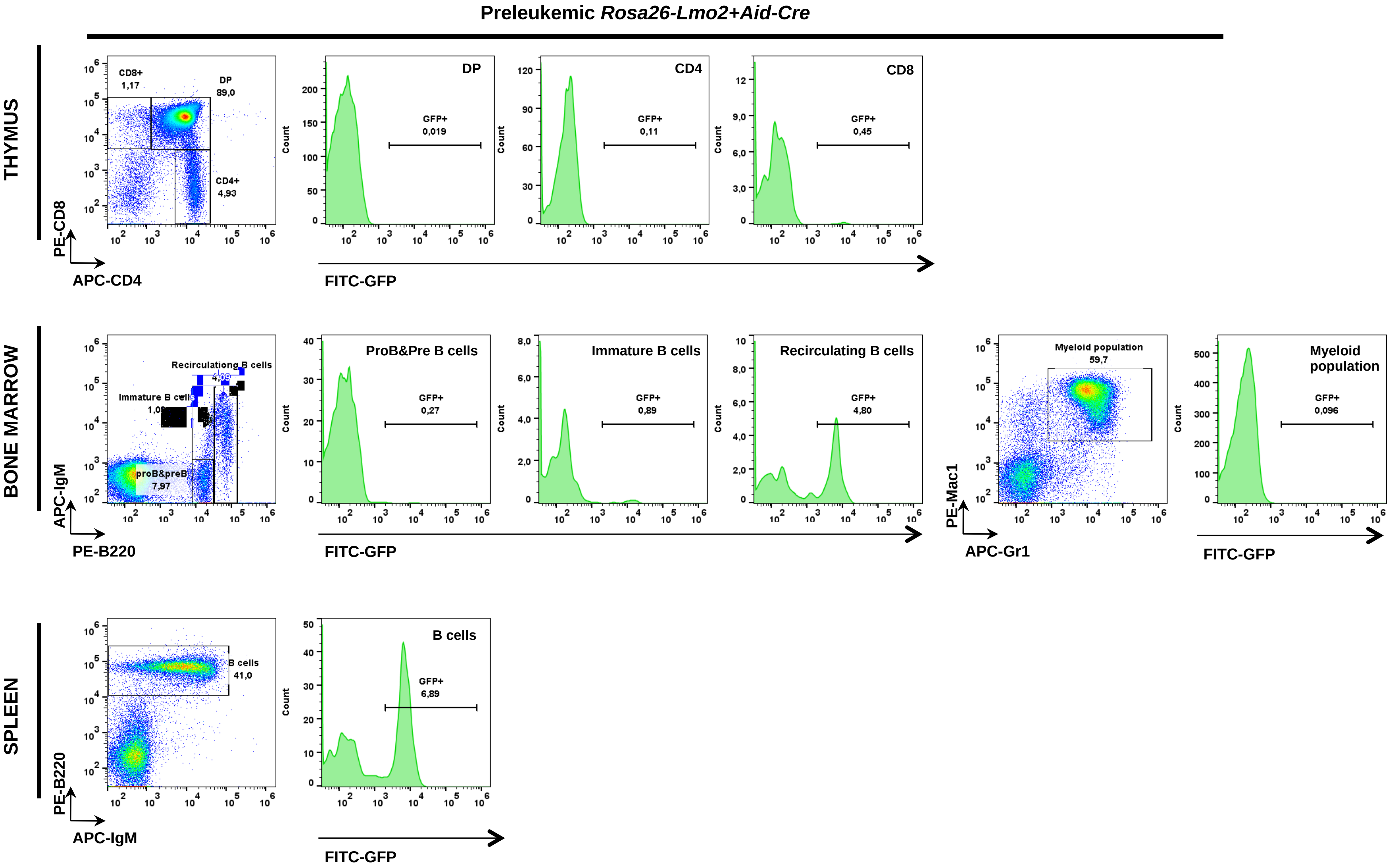

Appendix Figure S9

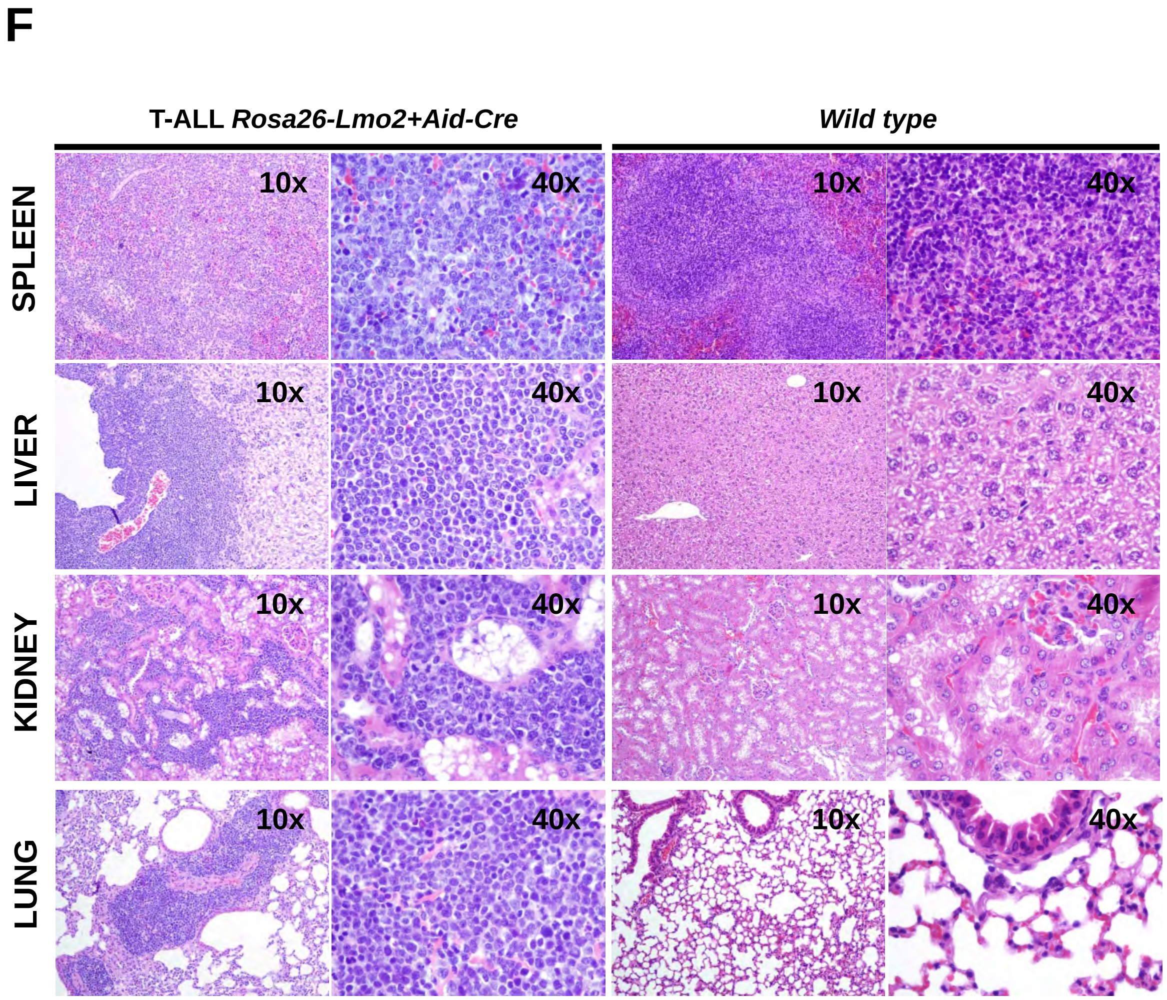

Supplement: Supplementary file 1 — Appendix [file EMBJ-37-e98783-s001.pdf]
