# Supplementary material for: Lmo2 expression defines tumor cell identity during T‐cell leukemogenesis
Source: EMBO J. 2018 Jun 7;37(14):e98783. doi: 10.15252/embj.201798783 (PMC6043907; doi:10.15252/embj.201798783)
Supplement: Supplementary file 5 — Table EV4 [file EMBJ-37-e98783-s005.pdf]

Table EV4: Trp53 mutations in different mouse models					
**Model**	**Mouse number**	**Mouse age at disease (months)**	**Cell of origin**	**CD4-CD8 phenotype**	**Trp53 SNV in mouse***
**Rosa26-Lmo2+Sca1-Cre**	C815	9.23	HSC	DP	c.719T>G; p.V212G
	K563	10.26	HSC	CD8+	c.198G>A; p.G238R
	K565	10.53	HSC	DP/CD8+	c.518C>A; p.P171H
**Sca1-tomato-IRES-Lmo2**	C120	12.67	HSC	DN	c.174T>C; p.Y230H
**Sca-Lmo2 $^{nu/nu}$ mice**	-	-	-	-	-
**Rosa26-Mmo2 + Mb1-cre**	W990	8.93	Pro-B cells	DP	c.211G>A; p.R24H
	K876	15.37	Pro-B cells	DP/CD8+	c.622C>T; p.P272S
**Rosa26-Lmo2 + Aid-Cre**	-	-	-	-	-
